# Aging Characterization and Preliminary Exploration of Gel-Based Cleaning of Cellulose Acetate in José Escada’s *Le Rituel*

**DOI:** 10.3390/gels11120954

**Published:** 2025-11-27

**Authors:** Susana França de Sá, Sara Babo, Artur Neves, Alexandra Garcia, Sofia Nunes, Aurora Cairoli, Maria João Melo

**Affiliations:** 1 LAQV-REQUIMTE, Department of Conservation and Restoration, NOVA School of Science and Technology, 2829-516 Caparica, Portugal; s.babo@fct.unl.pt (S.B.); afn.garcia@campus.fct.unl.pt (A.G.); aurora.cairoli@unive.it (A.C.); 2 The David Booth Conservation Center, The Museum of Modern Art, New York, NY 10019, USA; artur_neves@moma.org; 3 Department of Environmental Sciences, Informatics and Statistics, Ca’ Foscari University of Venice, 30123 Venice, Italy; 4 Department of Classics, Sapienza University of Rome, 00185 Rome, Italy

**Keywords:** plastics aging, plastics conservation, gel cleaning, contemporary art

## Abstract

Cellulose acetate (CA) is a semi-synthetic polymer widely present in modern and contemporary collections, yet its conservation poses major challenges due to its chemical and physical instability. Hydrolytic degradation, acetic acid release, plasticizer loss, and embrittlement compromise both structure and surface, making cleaning particularly difficult. Conventional cleaning methods may cause abrasion, extract additives, or alter gloss. Although hydrogels have shown promise for CA cleaning, the literature remains extremely limited. This study reports a preliminary investigation of gel-based cleaning on *Le Rituel* (1968), a heavily soiled cellulose acetate (CA) artwork by José Escada. The object’s condition was assessed through visual inspection, pH measurements, volatile acidity testing, and infrared spectroscopy. Cleaning tests were conducted on a CA replica (2006) with superficial soiling and on selected artwork areas. Two gel formulations were evaluated: the biopolymer agar-agar rigid gel and the synthetic viscoelastic poly(vinyl alcohol)-borax (PVAl-Borax) gel. Agar-agar was effective as a first step, reducing superficial soiling and humidifying adherent residues for subsequent removal, while PVAl-Borax was advantageous in the second step, as its viscoelastic properties enabled controlled mechanical action and facilitated the removal of more adherent residues. This case study demonstrates the potential of combined gel systems as versatile tools for CA conservation.

## 1. Introduction

Cellulose acetate (CA) is a semi-synthetic polymer that has played a central role in twentieth-century material culture, being widely used in cinematographic film, and modern and contemporary art [1,2,3,4]. Although CA holds significant historical, industrial, and artistic relevance, it is often regarded as one of the most unstable plastics in collections, with its degradation largely determined by environmental and production conditions [4]. Its degradation is characterized by acid-catalyzed hydrolysis of acetyl groups, releasing acetic acid in a process known as “vinegar syndrome,” which leads to embrittlement, dimensional changes, and plasticizer migration [5]. The molecular aging of CA artworks and films has been investigated by several authors such as Nunes et al. [2], who demonstrated that the degree of substitution (DS) is a structural parameter directly correlated with the extent of side-chain hydrolysis, where distinct conservation conditions match DS differences. It is also known that CA instability is exacerbated by surface soiling. Deposited dirt, or other residues, not only affect the artworks’ appearance but also act as hygroscopic agents that accelerate acid release and chain-scission [6,7]. Cleaning is therefore a critical yet challenging step in the conservation of CA.

Traditional dry or solvent-based methods can result in uncontrolled penetration, abrasion, extraction of additives, uneven gloss, or optical alterations [7,8]. In response, gel-based cleaning systems have recently gained increasing attention in cultural heritage conservation [8,9]. Hydrogels and organogels confine solvents or aqueous solutions within a polymeric network, allowing for localized delivery, minimal solvent penetration, and reduced mechanical stress [10,11]. Recent studies have demonstrated their efficacy for cleaning plastics, but systematic investigations on their application to CA artworks remain scarce [8,12]. In the POPART project (2008–2012), CA was one of the model plastics used to evaluate cleaning strategies [7]. Results indicated that detergent solutions applied with polyester microfiber cloths were effective and induced fewer scratches compared to dry cleaning methods. Building upon this, the NANORESTART project (2015–2018) assessed hydrogels—particularly PVAl formulations (Nanorestore Gel^®^ Peggy)—for their potential in cleaning CA and other plastics. Peggy gel provided a controlled release of cleaning fluids, reduced mechanical action, and allowed visual monitoring during treatment. Tests demonstrated that CA surfaces could be safely cleaned of both sebum and carbonaceous soils, with this hydrogel performing comparably or better than traditional tools while minimizing the risk of scratching and residue deposition. However, these studies [7,8] focused exclusively on new and colorless CA and evaluated only one type of PVAl-based hydrogel (Peggy). It is therefore important to expand research into other hydrogels that can also provide environmentally sustainable and practical solutions for conservators.

**Figure 1:**
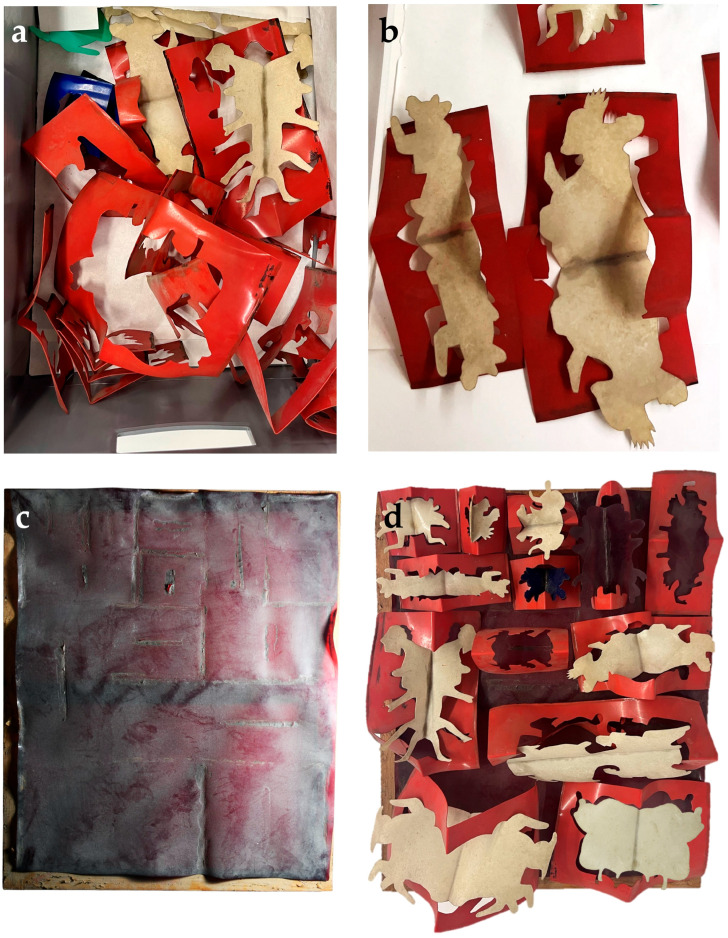
José Escada, *Le Rituel* (1968), cellulose acetate and wood. Condition of the artwork in 2025: (**a**) dismounted and completely detached CA modules, showing how they were conditioned; (**b**) detail of soiling on the CA modules; (**c**) detail of the CA base showing deformation, shrinkage, and dirt; and (**d**) proposed mounting layout illustrating missing CA modules.

This study reports, for the first time, the use of agar-agar and PVAl-Borax gels, applied both individually and sequentially, on a naturally aged, highly degraded, and heavily soiled cellulose acetate (CA) artwork—*Le Rituel* (1968) by the Portuguese artist José Escada (1934–1980) (Figure 1). The research hypothesis is that a combined gel-based approach can safely and effectively reduce surface soiling while preserving the fragile polymeric structure of the aged CA. The work focuses on a dismantled artwork considered lost due to its severe degradation, bridging the gap between laboratory studies and conservation practice. It provides a preliminary exploration of gel-based cleaning methods on naturally aged CA, emphasizing practical applicability. The results contribute to refining hydrogel-cleaning strategies for modern polymeric materials and support the development of accessible, sustainable, and safe cleaning procedures for the conservation of plastics-based artworks.

### José Escada and the Challenges of Cellulose Acetate Conservation

José Escada (1934–1980) studied Painting at the School of Fine Arts, where he met artists such as Lourdes Castro, René Bertholo, Gonçalo Duarte, and Costa Pinheiro, with whom he remained close throughout his career [13,14,15]. In the late 1950s, he moved to Paris on a Calouste Gulbenkian Foundation scholarship, joining the *KWY* group and exhibiting in its shows [14].

His early work combined abstraction and figuration, emphasizing biomorphic figures and geometric structures that formed a personal visual alphabet. He explored material qualities through watercolor and liquid fabric paint, achieving effects of near-impermanence.

By the mid-1950s, his focus on stains evolved into linear compositions, creating small, chromatically independent modules. Preferring paper over canvas for its lightness and flexibility, he developed three-dimensional reliefs through folding and cutting, producing silhouettes with concave and convex forms in contrasting colors [13].

This approach culminated in the *Relief Cutouts* series (1968), first exhibited at Galeria 111 in Lisbon [13]. In his final years, Escada turned to figurative painting with autobiographical themes [13,14,15]. Several of his reliefs from the 1960s, or “paintings-objects” as they were called, employed CA sheets, cut, folded, and glued in multiple colors to achieve complex visual depth. Some examples include *Relief orange* (1966), *La fête* (1967), *Dans la plage* (1968) and *Le Rituel* (1968), available in Supplementary Materials S1. These artworks are now in various stages of conservation and have been studied at NOVA School of Science and Technology [2,3,16].

In 2006, *Dans la plage* was the subject of an in-depth characterization, which included the production of replicas of one of the modules and a comprehensive conservation treatment. The artwork underwent surface cleaning with deionized water and the non-ionic surfactant Brij 700 (a polyethoxylated stearyl alcohol) [17], applied with cotton swabs. At the time, gel-based methods were not yet commonly employed in the conservation of plastics, and the level of soiling was low and most superficial. While the procedure was effective, its safety was not fully considered at the time, given the limited awareness of the potential risk of surface abrasion caused by the mechanical action of cotton swabs and embedded dirt particles. However, since the soiling was mostly superficial, abrasion risks were regarded as minimal.

In 2025, the need to clean *Le Rituel*—a heavily soiled, deformed, detached, and fully dismounted CA artwork—offered a valuable opportunity to evaluate the performance of gels. Hydrogels, particularly those derived from natural polysaccharides such as agar-agar, have gained recognition in conservation practice for their controlled release of cleaning agents, dimensional stability, and environmentally sustainable characteristics [9,18,19,20,21,22]. In parallel, the viscoelastic synthetic hydrogel based on PVAl-Borax provides tunable properties that can be adapted to specific conservation needs and supports the application of gentle mechanical actions, thereby enhancing cleaning efficiency [23,24,25,26]. These characteristics are particularly advantageous for cellulose acetate, whose chemical and physical instability requires controlled cleaning systems that minimize solvent exposure, mechanical stress, and additive extraction.

However, using these gels on CA artworks is rarely documented, and their potential for plastic heritage conservation still needs further investigation. This preliminary study examines the use of agar-agar and PVAl-Borax gels to clean a heavily soiled and deteriorated CA artwork, contributing to ongoing research in plastic conservation.

## 2. Results and Discussion

### 2.1. Artwork Condition

*Le Rituel* (1968) is a complex cellulose acetate (CA) assemblage composed of colored CA modules mounted on a CA dark red sheet and wooden strainer. Residual adhesive traces are present both on the sheet and on the modules. The cut-out positive modules are predominantly light beige, except for one blue and one dark red piece, all positioned along the central axis against red negative modules. Infrared analysis of the adhesive pointed to a degraded chloroprene-based adhesive. The planning of the mounting of the artwork revealed the loss of at least two elements.

The composition, while non-figurative, suggests organic silhouettes evoking hybrid human–animal forms, reinforcing the metaphorical resonance of the title *Le Rituel*. Comparable structural strategies were identified in Escada’s other “pintura-objeto” works, such as *Relief Orange* (1966), *La Fête* (1967), and *Dans la Plage* (1968).

**Figure 2:**
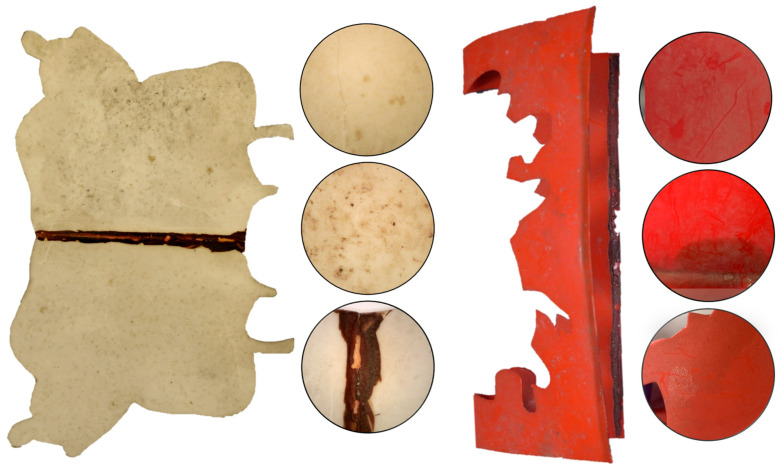
Details of *Le Rituel* superficial and ingrained dirt and dust: beige (**left**) and red (**right**) CA modules.

Macroscopic observation revealed that the artwork is in a highly fragile state, requiring careful handling. Both superficial and ingrained dirt and dust are visible across the surface (Figure 1 and Figure 2). The soiling appears predominantly carbonaceous (Figure 1), and two main types of stains can be distinguished: light yellowish deposits, and darker, smaller spots (Figure 2). These deposits have altered the overall chromatic and glossy appearance of the object and are feeding the hydrolytic aging pathways [6]. Additionally, some cracks and microcracks are visible in both beige and red modules, further underscoring the structural fragility of the piece.

On the central axis of the verso of the beige modules and on the limits of the red modules, hardened dark brown residues of the adhesive are clearly visible (Figure 2). Beneath these residues, a salmon-pink discoloration of the polymer can be observed, especially in the beige elements. This chromatic alteration can be attributed to the interaction of the adhesive solvent and to the degradation of the CA/adhesive layer. As noted by Nunes [3], the photooxidation of the adhesive residues will generate chlorine radicals. These reactive species attack the CA structure, for example by abstracting hydrogen atoms and producing hydrochloric acid (HCl). The formation of HCl decreases the pH at the polymer surface and accelerates acid-catalyzed hydrolysis of the acetyl groups.

To assess the possible presence of active deterioration through the release of acidic volatiles, free acidity indicator strips were employed. The release of acetic acid was indicated by the color change of the strip from dark blue to bluish green, pointing to values of 5. This indicates that chemical degradation of CA elements has already begun and is likely to intensify over time, even though the vinegar-like odor was still not clearly detected. pH measurements were also conducted in selected areas of the artwork. Determined values in CA areas were around 6, whereas adhesive areas were around 5, slightly more acidic.

These results are aligned with the acid hydrolysis reported in the literature [2,3,27]. In its initial stage, hydrolysis involves the cleavage of the side chains of cellulose acetate, which are replaced by hydroxyl groups, accompanied by the release of acetic acid. At more advanced stages, scission of the main polymer chains may also occur. The release of acetic acid produces a characteristic vinegar-like odor, which is why acid hydrolysis of cellulose acetate is also referred to as the “vinegar syndrome” [28]. As this odor was not clearly detected in the artwork under study, it is suggested that its chemical degradation is still at an early stage. However, other visible forms related to the material decay are clearly found in the artwork.

Deformations, shrinkage, and loss of flexibility are visible in *Le Rituel*, which may occur because of plasticizer depletion [5,28,29]. Both CA modules and support sheet already lost flexibility, becoming stiffer and more brittle, and therefore exhibiting fragility to the touch. Deformation and shrinkage are also evident, particularly at the edges of the CA modules.

**Figure 3:**
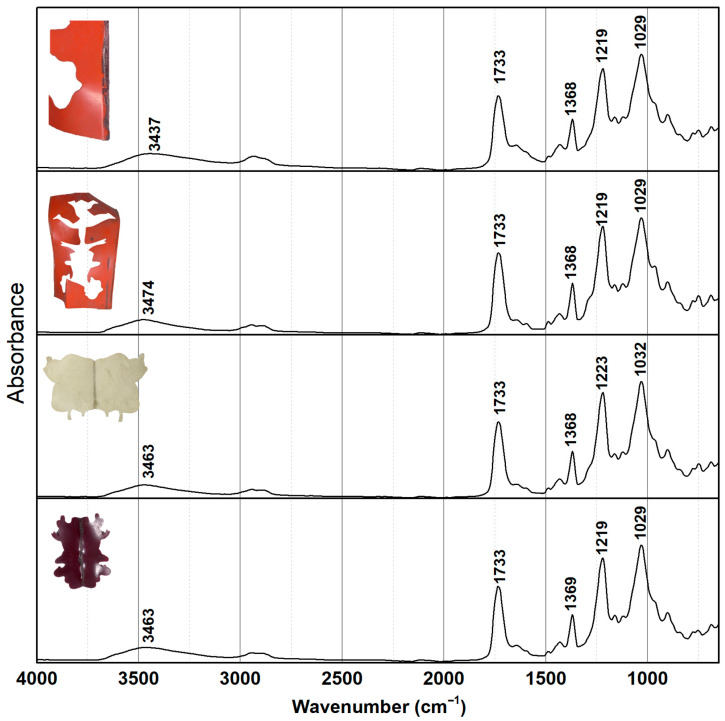
ATR-FTIR spectra of CA modules from *Le Rituel* divided by CA color: dark red, light beige, red and red areas close to adhesive layer (from **bottom** to **top**).

The average degree of substitution (DS) values obtained by FTIR-ATR highlight distinct patterns of chemical stability across the different cellulose acetate (CA) elements in *Le Rituel* (Figure 3 and Table 1). The red modules (with c. 0.8 mm thickness) near to the adhesive residues present the lowest DS values (1.71 ± 0.19). This indicates significant hydrolysis of the acetyl groups, consistent with the deterioration observed in these areas. The variability of the data (high standard deviation) suggests heterogeneous degradation, with some areas more affected than others. This supports the hypothesis that adhesive residues act as localized catalysts of CA degradation. In contrast, the red modules distant from the adhesive retain a much higher DS (2.12 ± 0.06), very close to the values observed for the beige modules (2.10 ± 0.10), with c. 0.82 mm thickness. The narrower variability indicates a more homogeneous and stable condition. The similar conservation condition between these areas indicates that intrinsic factors, namely the colorants, did not play a major role in accelerating decay here. On the other hand, the dark red module (with c. 0.55 mm thickness) shows a lower DS and higher standard deviation (2.00 ± 0.22), meaning that intrinsic factors might be involved. The heavy soiling across the entire work may also have contributed to varying deterioration levels in the CA sheets, which could explain the higher standard deviation values. For more details on the DS calculation and vibration or stretching associated with each infrared peak, please refer to Supplementary Material S2.

### 2.2. Naturally Aged Replica

Replicas from one of the modules of *Dans la Plage* were produced in 2006 with similar materials to the original piece: a medium density fiberboard (MDF) support measuring 19.5 × 18 × 0.5 cm; cellulose acetate sheets from Mazzuchelli (yellow and orange sheets with 0.6 mm thickness, white sheet with 1.1 mm thickness); a cyanoacrylate-based adhesive and a chloroprene-based contact glue, both by Pattex. These replicas have been subjected to natural aging in indoor non-controlled environmental conditions for almost 20 years and present several alterations, similar to the ones found in the original artworks. Besides superficial dust, the CA sheets from the replica showed deformations, loss of flexibility, and significant shrinkage due to degradation. For these reasons, they constitute perfect samples for assessing aging but also for testing treatment procedures. The DS values for the CA replica were also calculated and values around 2.70 ± 0.2 were determined, which could be related to a more recent production date and absence of heavy soiling in the last 20 years. ATR and Raman spectra in the recent study of the replica can be assessed in Supplementary Material S2 and S3.

### 2.3. Cleaning Tests in Cellulose Acetate Replica and Artwork

The confirmed fragile and unstable condition of *Le Rituel* led to the importance of removing the overall soiling based on aqueous methods, and particularly, using gels.

Four different cleaning tests were carried out on the CA replica’s module from 2006 to assess safety (as mostly superficial dirt was present) and on selected areas from the artwork to assess both safety and efficacy. These areas were selected as they represented the overall dirt and condition of the entire artwork, as shown in Figure 2.

The selected tests included agar-agar at 2% and 4%, and PVAl-Borax (alone, and with Tween 20 at 1%). The use of ethanol, either in solution or in gel form, was discarded, as it could compromise the safety of the object. The Hansen solubility parameters for ethanol (12.7) and cellulose acetate (11.4) show that these values are too close, and it must be considered that the polymer tends to become more polar with aging. Tween^®^ 20, a non-ionic surfactant, was selected for its mild action, good solubility, and low foaming properties. Compared to other surfactants commonly used in conservation (e.g., Triton X-100), Tween 20 offers reduced toxicity and is less prone to leave residues on treated surfaces, making it particularly suitable for aqueous cleaning systems in heritage conservation. Its application in the field has been discussed by [30,31].

With regard to agar-agar, both concentrations tested were effective in removing the replica’s superficial dirt, and no negative alterations of the surface were observed. As expected, however, this hydrogel proved ineffective against residues of non–water-soluble soiling or in more persistent soil due to the limited contact between the agar sheets and the CA surface. Also, the 2% agar-agar gel left more water on the material (less retentive) and showed a greater tendency to leave gel residues when handled, as it disintegrated more easily; therefore it was discarded for further tests. Cleaning with the partially disintegrated 4% gel, applied with a rubber brush, aided the removal of more encrusted dirt and highlighted the need for some degree of mechanical action. However, the additional requirement to remove all gel residues made the method more time-consuming and more prone to abrasion. Furthermore, this cleaning method did not produce a homogeneous surface appearance, as certain areas remained cleaner than others. To achieve complete dirt removal, the agar-agar gel had to be complemented with other cleaning methods.

**Figure 4:**
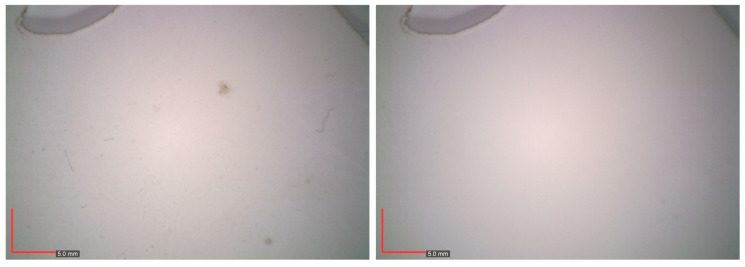
Before (**left**) and after (**right**) CA replica’s surface cleaning with the combination of agar-agar 4% rigid gel and PVAl-Borax viscoelastic gel, followed by localized cleaning of PVAl-Borax loaded with Tween 20 at 1% of embedded stains of soil.

Unlike agar-agar, which forms a rigid gel, PVAl-Borax^®^ is pliable and can therefore be applied to irregular surfaces while incorporating mechanical action. This type of hydrogel exhibits high water retention capacity and consequently low surface wettability, which enabled a controlled, safe, and effective cleaning of the surface. Although PVAl-Borax gel is known to liquefy (lose its physical crosslinking) when in contact with acidic surfaces, the pH of the artwork proved to be within a safe range for its use. When tested as a hydrogel with distilled water alone, it proved effective lighting more persistent stains, especially when used after the agar-agar gel application. The addition of 1% of the surfactant Tween20^®^ also yielded good results, although a second cleaning step (clearance) was required to ensure surfactant removal. The combination of these methods proved safe and effective for removing more embedded soiling stains in the replica (Figure 4), although the degree of soiling in the replicas was significantly lower than that observed on the artwork.

**Figure 5:**
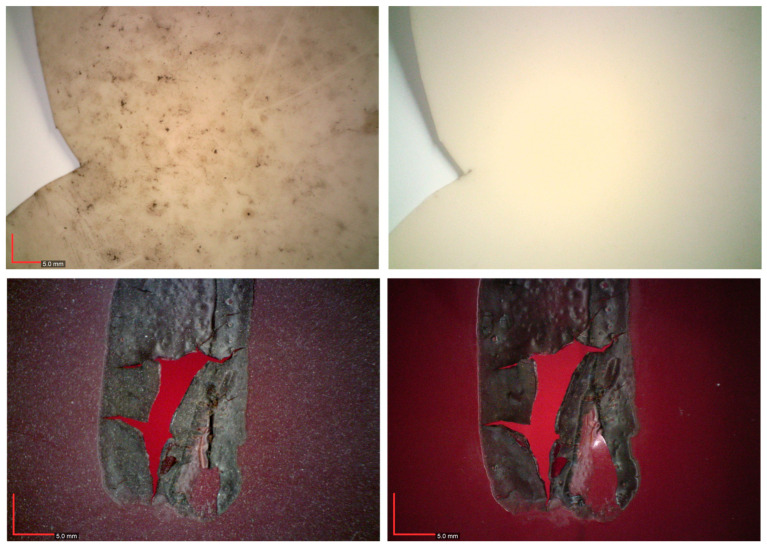
Before (**left**) and after (**right**) cleaning of Le Rituel areas in CA light beige and dark red CA modules with the combination of agar-agar 4% rigid gel and PVAl-Borax viscoelastic gel.

**Figure 6:**
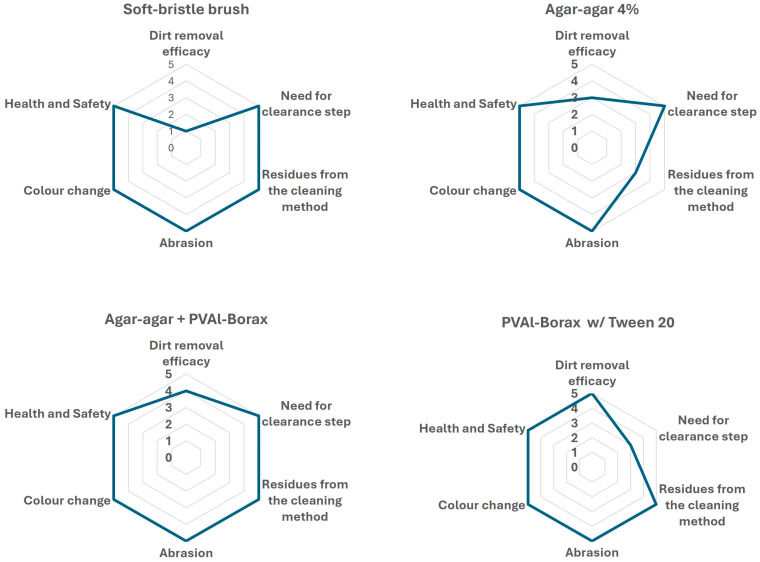
Star diagrams showing the visual assessment for each cleaning method tested on the artwork. Scores range from 0 (poor performance/high risk) to 5 (optimal performance/low risk). Higher values indicate better overall results, meaning greater cleaning efficacy, reduced risks (abrasion, color change, residues), no clearance step required, and increased health and safety.

Based on these preliminary results, a stepwise protocol for testing the removal of superficial and adherent soiling is proposed for the artwork. First, a soft-bristle brush was combined with an air blower to remove loose surface particles; however, the efficiency of this step was very low. Next, a 4% agar-agar rigid gel was applied for 5–10 min, with repetitions, when necessary, followed by PVAl–Borax gel to homogenize the cleaning, and slight water residues persisted on the surface due to its lower retention capacity when compared to PVAl-Borax. The subsequent application of PVAl–Borax gel enhanced cleaning efficiency and provided a more uniform result. For heavily soiled areas, the sequential use of agar-agar gel followed by PVAl–Borax was significantly more effective than PVAl–Borax alone, as the agar-agar step, despite its lower retention capacity, was particularly effective in softening and slightly lubricating the soiling, thereby facilitating its removal. In areas with greasy deposits or stains, localized application of PVAl–Borax gel loaded with Tween20^®^ surfactant was effective, though it required a clearance step to remove surfactant residues. Representative before- and after-cleaning images are shown in Figure 5. Figure 6 presents star diagrams illustrating the visual assessment criteria for each method assessed in the artwork.

**Figure 7:**
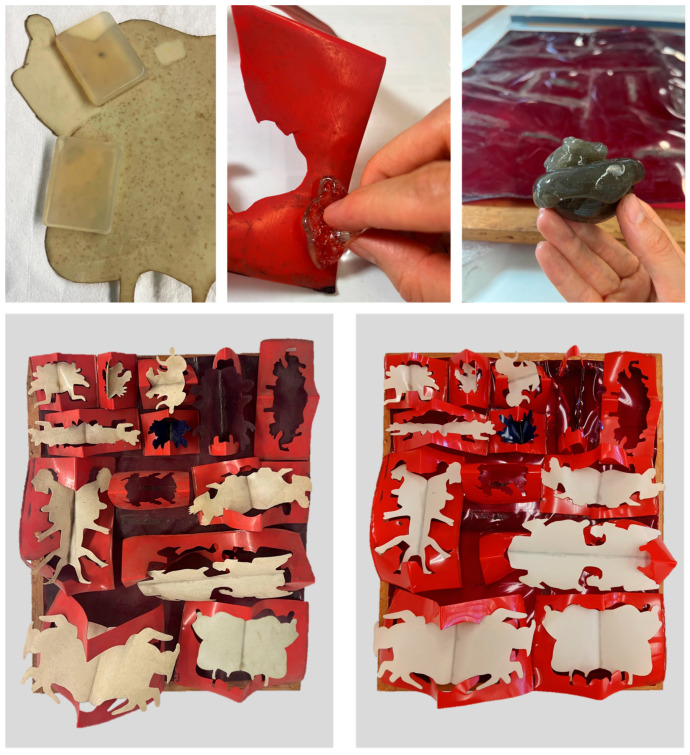
Cleaning of *Le Rituel*. (**Top**): images representing the application of agar-agar rigid gel (**left**) and PVAl–Borax viscoelastic gel (**center** and **right**) on beige and red modules to remove soiling and stains. (**Bottom**): overall views of the artwork before (**left**) and after (**right**) cleaning, evidencing the recovery of surface gloss and chromatic contrast.

From these results, the entire artwork was cleaned (Figure 7). The overall cleaning of *Le Rituel* demonstrated that the combined agar-agar 4% and PVAl-Borax^®^ systems provided a safe and effective reduction in surface soiling, restoring the chromatic contrast of the CA modules and significantly improving the visual legibility of the composition.

## 3. Conclusions

This study demonstrates the potential of hydrogel systems for the cleaning of cellulose acetate artworks, a class of heritage objects notoriously difficult to treat due to their chemical instability and mechanical fragility. The preliminary cleaning trials conducted on a sacrificial CA replica pointed to the safety of agar-agar and PVAl-Borax formulations, which were subsequently applied to the case study artwork *Le Rituel* (1968) by José Escada (Figure 7).

Agar-agar gel at 4% concentration proved effective for the removal of superficial soiling, although complementary methods were required to homogenize cleaning across the surface. PVAl-Borax gel, owing to its pliability and adaptability to irregular geometries, facilitated the reduction of more adherent residues and, when combined with Tween 20, improved the removal of embedded soiling, albeit requiring subsequent rinsing. The combination of these gels in a stepwise protocol resulted in a significant aesthetic improvement of the artwork while preserving its fragile material integrity. The degraded chloroprene-based adhesive will need further evaluation to determine if it remains stable or if other problems need to be addressed.

Beyond this specific case, the results highlight the importance of tailoring gel formulations to both the degradation state of CA and the nature of soiling present. They also underscore the necessity of integrating mechanical action and sequential cleaning strategies to achieve satisfactory outcomes. It also suggested that areas with lower DS, indicative of more advanced hydrolysis, showed higher sensitivity during cleaning, especially to mechanical action, highlighting the importance of adapting cleaning procedures accordingly.

As this study represents preliminary, qualitative exploration based on a single artwork, further research is recommended to evaluate the long-term effects of PVAl-Borax and agar-agar gels on CA cleaning through artificial aging experiments, to investigate cleaning performance at the micro- and molecular scales, and to expand the range of safe and cost-effective alternatives for large-scale cleaning treatments.

Despite these limitations, the results provide a practical foundation for future systematic studies and large-scale conservation treatments involving aged CA surfaces.

Overall, this study contributes new insights into gel-based cleaning strategies for CA artworks and supports their broader application in the conservation of modern and contemporary plastics heritage.

## 4. Materials and Methods

### 4.1. Gels: Preparation and Application

Agar-agar, PVAl and Borax were purchased from AN.T.A.RES (San Lazzaro di Savena, Italy). Agar-agar is a biopolymer and a physical rigid gel based on a polysaccharide extracted from red algae. It is slightly yellow, semi-transparent, completely non-toxic, and easily available. Agar-agar rigid physical gel was prepared at 2 and 4% in water, following the recipe described in [32]. PVAl-Borax moldable gel is a synthetic physical gel that was prepared at 8%, following the recipe described in [33,34]. The hydrogel is transparent, non-adhesive, and highly malleable, enabling close contact with surface irregularities under minimal pressure. For more persistent soiling, PVAl-Borax gel was loaded with 1% of Tween 20 (Kremer Pigmente GmbH & Co. KG, Aichstetten, Germany). Polysorbate 20 (Tween^®^ 20) is a non-ionic surfactant commonly used in conservation for aqueous cleaning systems, owing to its mild action, good solubility, and ability to reduce surface tension without leaving significant residues.

Rigid agar–agar gel sheets were applied to the CA surfaces for 5–10 min and then removed using plastic or rubber spatulas. PVAl–borax gel was employed in a malleable form, applied with gentle finger pressure to provide a massaging mechanical cleaning action. Once the transparent gel became loaded with soil, it was replaced with a fresh portion.

### 4.2. Examination and Analytical Instrumentation

The condition of the artwork was assessed by visual examination, optical microscopy, and A-D Strips^®^ (Dancan Cine Film Service, SL, Barcelona, Spain) to detect volatile acetic acid release. One CA module was sealed in a barrier film bag with A-D strips for two days, with one control strip maintained in another similar sealed environment but without the artwork for comparison purposes. Surface pH was measured using Nahita universal indicator paper strips (Auxilab, Beriáin, Spain) with distilled water application (one drop). Measurements were taken at different locations on the CA modules and in the adhesive areas.

Comprehensive photographic documentation under visible light, including raking light examination was carried out to reveal surface irregularities and deposit patterns. Digital imaging before and after cleaning was carried out with the portable digital optical microscope Dino-Lite AM73115MTF.

Infrared spectroscopy in attenuated total reflection mode (ATR-FTIR) was carried out using the handheld Agilent 4300 spectrophotometer (Agilent, Santa Clara, CA, USA), equipped with a ZnSe beam splitter, a Michelson interferometer, and a thermoelectrically cooled DTGS detector. Spectra between 4000 and 650 cm^−1^ were acquired with a diamond ATR module, 128 scans and 4 cm^−1^ resolution. Background spectra (air) were collected between every acquisition. The degree of substitution of cellulose acetate was calculated using the FTIR-ATR calibration curve developed by [2].

### 4.3. Cleaning Assessment

All cleaning tests were visually evaluated according to six criteria: cleaning efficiency (visible removal of soiling), presence of residues (from gels or liquid agents), surface alterations including abrasion and color change, possible need for clearance, and safety and health. Each criterion was rated from 0 (poor performance/high risk) to 5 (optimal performance/low risk). The results were synthesized in star diagrams following the comparative visual evaluation method established by Bartoletti et al. [35]. This approach allows for comparison of multiple systems and visualizes both performance and visually perceivable risk factors. The most promising systems were subsequently applied to the entire artwork in a stepwise manner.

## Figures and Tables

**Table 1:** Average degree of substitution and standard deviation for each module analyzed.

Module	Average DS	Standard Deviation
Red, near adhesive	1.71	0.19
Red, far from adhesive	2.12	0.06
Beige	2.10	0.10
Dark Red	2.00	0.22

## Data Availability

All data are available in the current paper and in the Supplementary Materials.

## Supplementary Materials

The following supporting information can be downloaded at: https://www.mdpi.com/article/10.3390/gels11120954/s1, Figure S1: Front and rear views of *Relief orange* (1966); Figure S2: Front and rear views of *La fête* (1967); Figure S3: Front and rear views of *Dans la plage* (1968); Table S1: Average and standard deviation of the degree of substitution for both faces of each sample analyzed from infrared spectra. For more details, see SM2; Figures S4–S12. ATR-FTIR spectra of the ten spots analyzed on each sample (orange 1–3, white 1–3, yellow 1–3). For each sample, the upper plot shows the spectra of the five spots on one face, and the lower plot shows the spectra of the five spots on the opposite face. Table S2. Degree of substitution for each point analyzed. Figure S13. Raman spectra of reference materials: cellulose triacetate (TCA), diethyl phthalate (DEP), triphenyl phosphate (TPP), and titanium dioxide (TiO_2_). Figure S14. Raman spectra of orange (red), yellow (orange), and white (black) cellulose acetate sheets obtained with Mira. Acquisition times: 5 s (orange), 8 s (yellow), 10 s (white). Table S3. Orange, yellow, and white sheets with their attributions, compared to reference spectra. Figure S15. Raman spectra of an orange replica of Dans la plage (micro-Raman, 15 s acquisition time). Table S4. Orange cellulose acetate sheet compared to reference spectra of cellulose acetate and diethyl phthalate. Scheme S1. A piece from the artwork Dans la plage that was used to prepare the replica with Mazzuchelli sheets. Photos by Sara Babo. References [36,37,38] are cited in the Supplementary Materials.

## Abbreviations

The following abbreviations are used in this manuscript:

CA	Cellulose acetate
PVAl	Poly(vinyl alcohol)
CR	chloroprene
