# Supplementary material for: Aging Characterization and Preliminary Exploration of Gel-Based Cleaning of Cellulose Acetate in José Escada’s Le Rituel
Source: Gels. 2025 Nov 27;11(12):954. doi: 10.3390/gels11120954 (PMC12732853; doi:10.3390/gels11120954)
Supplement: Supplementary file 1 [file gels-11-00954-s001.zip › gels-3925548-supplementary.pdf]

Supplementary Materials S1. Data on the pieces studied by José Escada S

S1.1 *Relief orange*

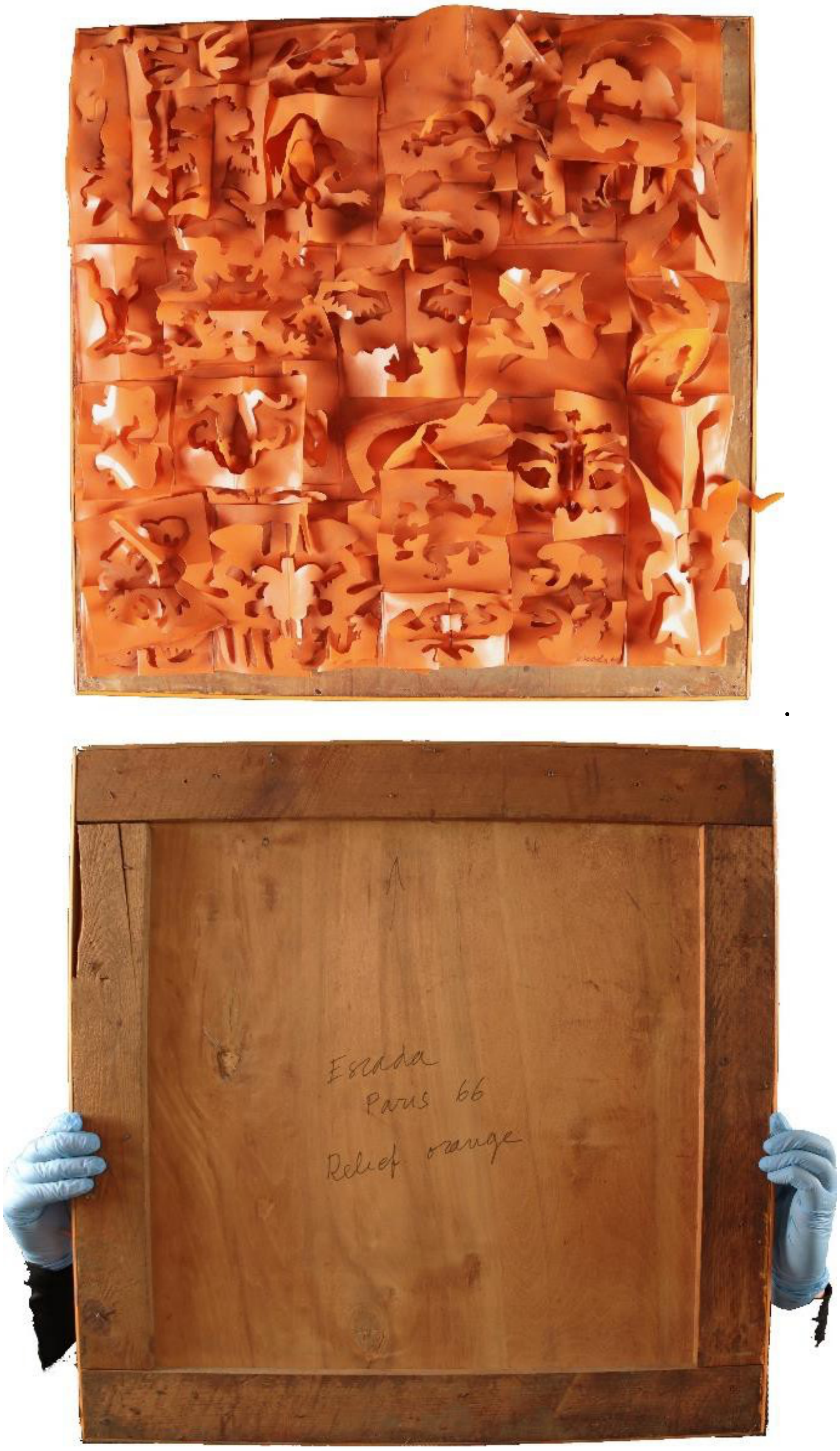

Figure S1. Front and rear views of *Relief orange* (1966).

Information

Owner: Department of Conservation and Restoration

Provenance and context of study: Before arriving at the Department, the work under study belonged to Rui Mário Gonçalves and, reportedly, was given to him by the artist himself. Thus, after being produced by Escada in Paris in 1966, it was transported to Portugal and remained in the critic's home until 2006. There are no records in catalogs or books of the work's presence in exhibitions. In 2006, it was loaned to the department, where it remains to this day. In the 2017/2018 academic year, it was studied and characterized in the Analytical Methods for Cultural Heritage course and is currently the central subject of study in this master's thesis, which focuses on the degradation of cellulose acetate.

Diagnostic Start Date: April 20, 2018 *End Date:* September 23, 2019

Current Location: Department of Conservation and Restoration at Nova School of Science and Technology, Painting Laboratory Reserve

***Relief Orange* by José Escada (Lisbon, 1934-1980), 1966**

Category: According to the artist, this type of work falls into the "Object Painting" category. Dimensions (max. height x max. width x max. depth): 57 x 56.7 x 7 cm

Description: Sculptural piece on a quadrangular panel with a vertical orientation, created by cutting and gluing orange plastic sheets in relief onto an orange base of the same material, previously placed on a panel with orange painted sides. The panel is shaped like a quadrangular prism with rounded corners, with sides painted in opaque orange. Its front face supports the sculpture, composed of orange plastic sheets. This plastic sheet, which nearly covers the entire panel, is nailed and glued in place. A total of 28 modules are glued to the sheet. These are rectangular sheets of various sizes, folded and cut into organic, abstract, and somewhat anthropomorphic or animalistic forms. The negative and positive shapes are arranged together with opposite folding directions. The vertical orientation of the work is clearly reflected in the direction of the inscriptions.

Previous interventions: There are no records of previous interventions.

Registration: Escada 66 in one of the modules of the work

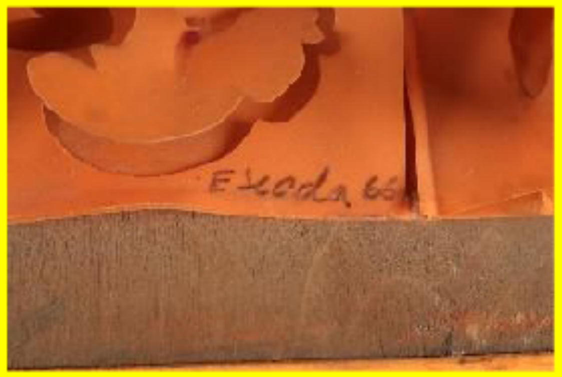

S1.2 *La fête*

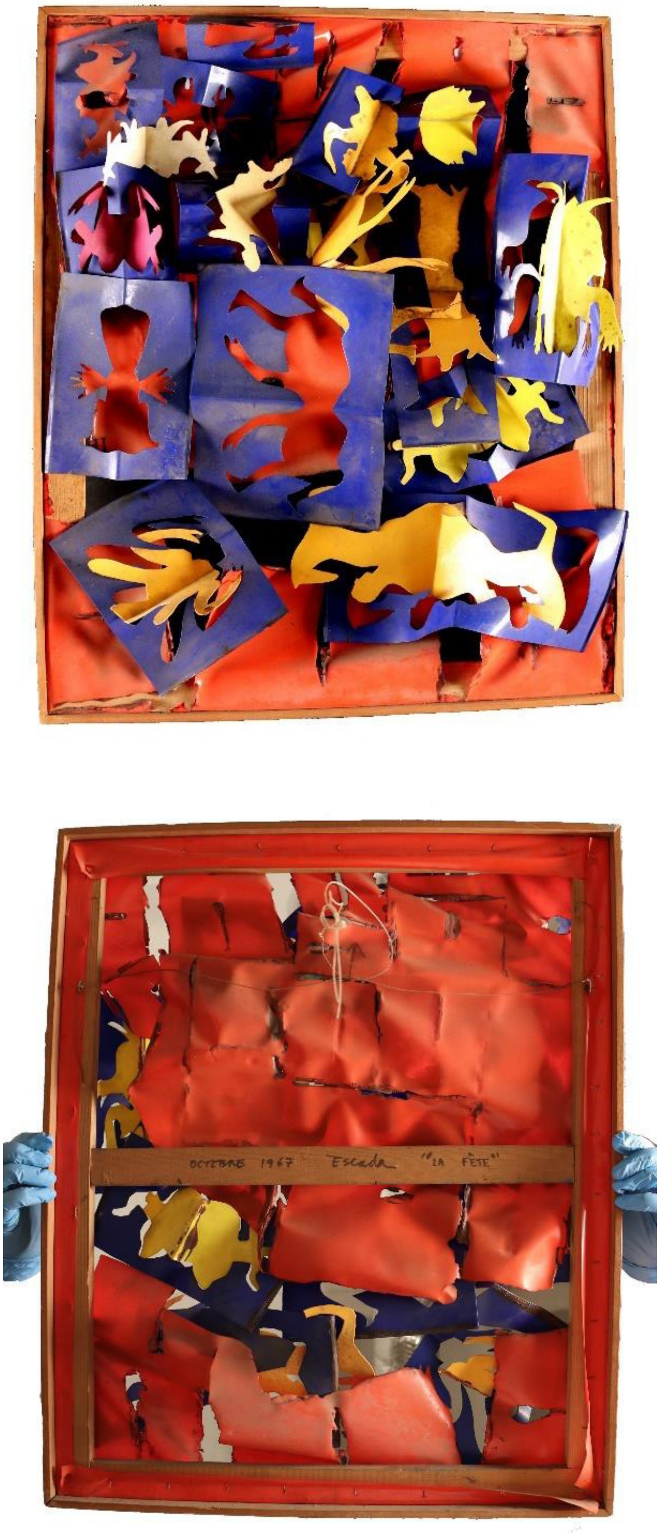

Figure S2. Front and rear views of *La fête* (1967).

Information

Owner: Maria Arlete Alves da Silva (manager *Galeria 111*)

Provenance and context of study: According to owner Maria Arlete Alves da Silva, this work, after being produced in Paris in 1967, was transported to Portugal directly to Galeria 111. She claims that the work arrived unfit for exhibition or sale due to its precarious condition. Thus, it remained in the Gallery's reserves until April 20 of this year, when it was transported to the Department of Conservation and Restoration for study as part of this master's thesis.

Diagnostic Start Date: April 24, 2019 *End Date: September 23, 2019*

Current Location: Department of Conservation and Restoration at Nova School of Science and Technology, Painting Laboratory Reserve

***La fête* by José Escada (Lisbon, 1934-1980), 1967**

Category: According to the artist, this type of work falls into the "Object Painting" category. Dimensions (max. height x max. width x max. depth): 62.5 x 52 x 3.5 cm

Description: A rectangular sculptural piece on a vertically oriented panel, created by cutting and gluing plastic sheets in relief, using primary colors yellow and blue, onto a red base of the same material. The base was previously mounted on a wooden grid with a structure similar to that of painting canvases. The panel is shaped like a rectangular prism. Its front face supports the sculpture with plastic sheets of various colors: yellow, blue, white, purple, and green. The plastic sheet, which nearly covers the entire grid, is nailed and glued to it. Sixteen modules, totaling 29, are attached to the sheet. These are sheets with rectangular shapes of various sizes, folded and cut into organic, abstract, and somewhat anthropomorphic or animalistic forms. The negative and positive shapes are arranged together with opposite folding directions. The work's vertical orientation is clearly indicated by the inscription.

Previous interventions: There are no records of prior interventions; however, given the presence of adhesive in the work (possibly a chloroprene-based adhesive), as this material only began to be commercialized on a large scale in the 1960s, the hypothesis of a later intervention can be raised.

S1.3 Dans la plage

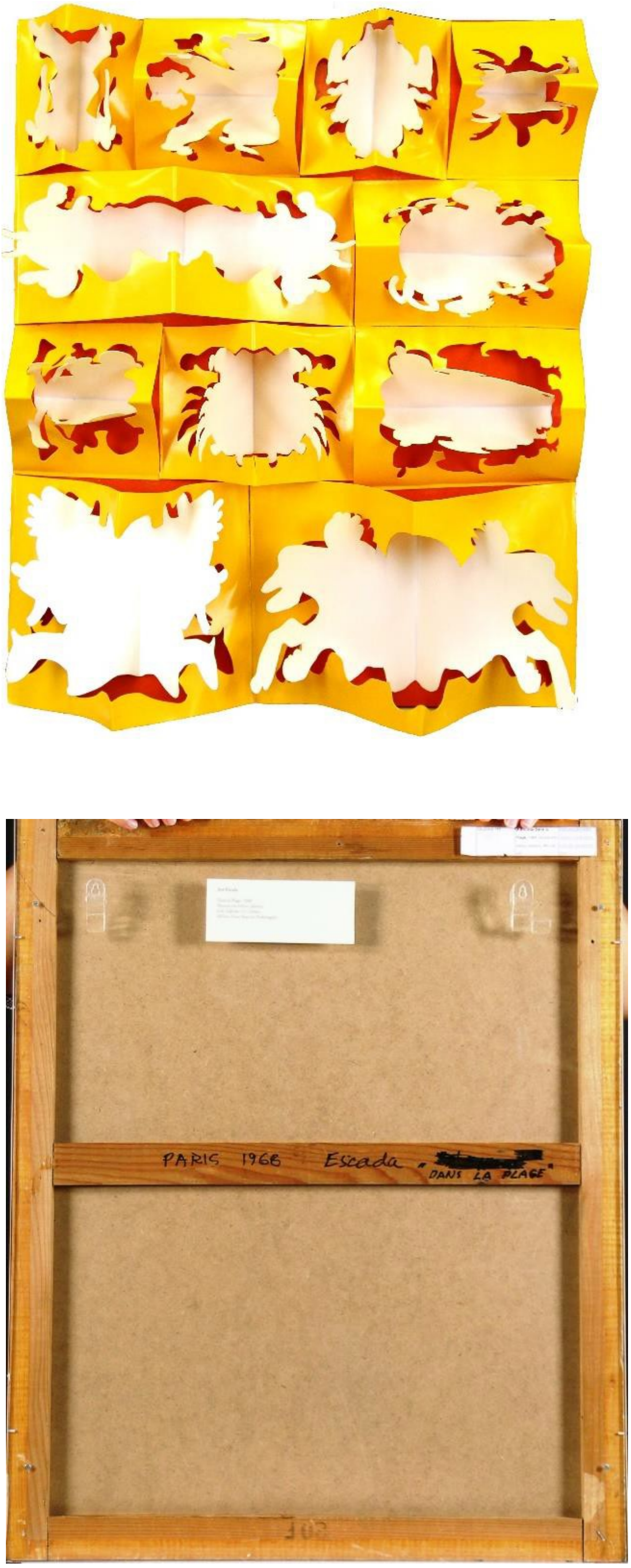

Figure S3. Front and rear views of *Dans la plage* (1968).

Information

Owner: National Museum of Contemporary Art of Chiado. In Portuguese, Museu do Chiado – Museu Nacional de Arte Contemporânea (IPM).

Provenance and context of study: This work was studied in 2006 by Dr. Sara Babo. It initially belonged to Galeria 111 and was acquired by the State in 2001, during the KWY exhibition at the Belém Cultural Center, and has since become part of the collection of the Chiado Museum – MNAC, nº2574. To date, there is no record of the work having been exhibited or reproduced. Since 2001, in addition to the aforementioned exhibition KWY. Paris 1958-1968, at the Belém Cultural Center, the work has been exhibited at the Chiado Museum in the exhibitions New Acquisitions and Donations 2000-2001, from 2001 to 2002, and Difference and Conflict. The 20th Century in the Collections of the Chiado Museum, in 2002; in 2003, it was shown in the exhibition 1960-1980, Years of Artistic Normalization in the Collections of the Chiado Museum at the Francisco Tavares Proença Júnior Museum in Castelo Branco. The work was cited and reproduced in the catalogs of the respective exhibitions. In this study, it will be a fundamental parallel.

Diagnostic Start Date: October, 2005 *End Date:* October, 2006

Current Location: National Museum of Contemporary Art of Chiado. In Portuguese, Museu do Chiado – Museu Nacional de Arte Contemporânea.

***Dans la Plage* by José Escada (Lisbon, 1934-1980), 1968**

Category: According to the artist, this type of work falls into the "Object Painting" category. Dimensions (max. height x max. width x max. depth): 69 x 57 x 12 cm

Description: A rectangular, vertically oriented "object-painting," created by cutting and gluing a yellow and white plastic sheet in relief onto an orange base made of the same material. This abstract composition is built from symmetrical modules of organic forms that have figurative suggestion, each independent in shape, size, and orientation of their axes of symmetry (vertical or horizontal). The piece features 11 modules cut from sheets of a plastic material identified as cellulose acetate, each consisting of two parts: a positive and a negative, colored white and yellow respectively. Each module has an axis of symmetry (vertical or horizontal) aligned with the folding line of the sheet, Figure S3. These modules are glued onto an orange sheet of the same material, which is then attached to the support—a medium-density fiberboard (MDF) panel supported by a wooden grid. The work is enclosed in a clear acrylic box, with its base screwed onto the wooden grid. The diagram and figure below illustrate the composition of the piece.

Previous interventions: The work was restored for the 2001 exhibition, as mentioned in the KWY exhibition catalogue.

Registration: Paris 1968 Staircase "DANS LA PLAGE" below another erased name, in black pen, on the central crossbar of the grid. Pencil numbering in the corner of each module (from 1 to 22, starting in the upper left corner, odd numbers on the white pieces, even numbers on the yellow pieces).

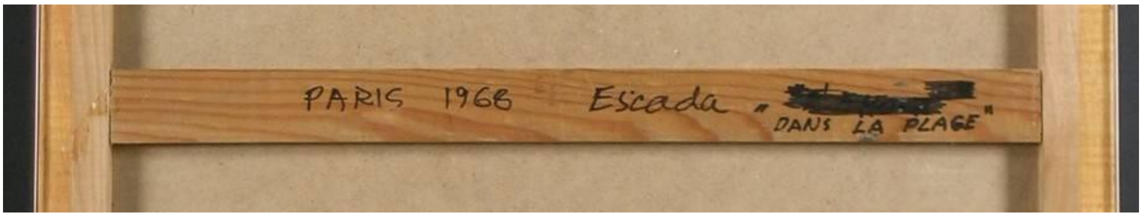

Supplementary Materials S2.

ATR-FTIR data on Mazzuchelli sheets selected in 2006 to prepare a replica of *Dans la plage* by José Escada. An orange, white, and yellow pieces were studied. For more details, please see Scheme S1.

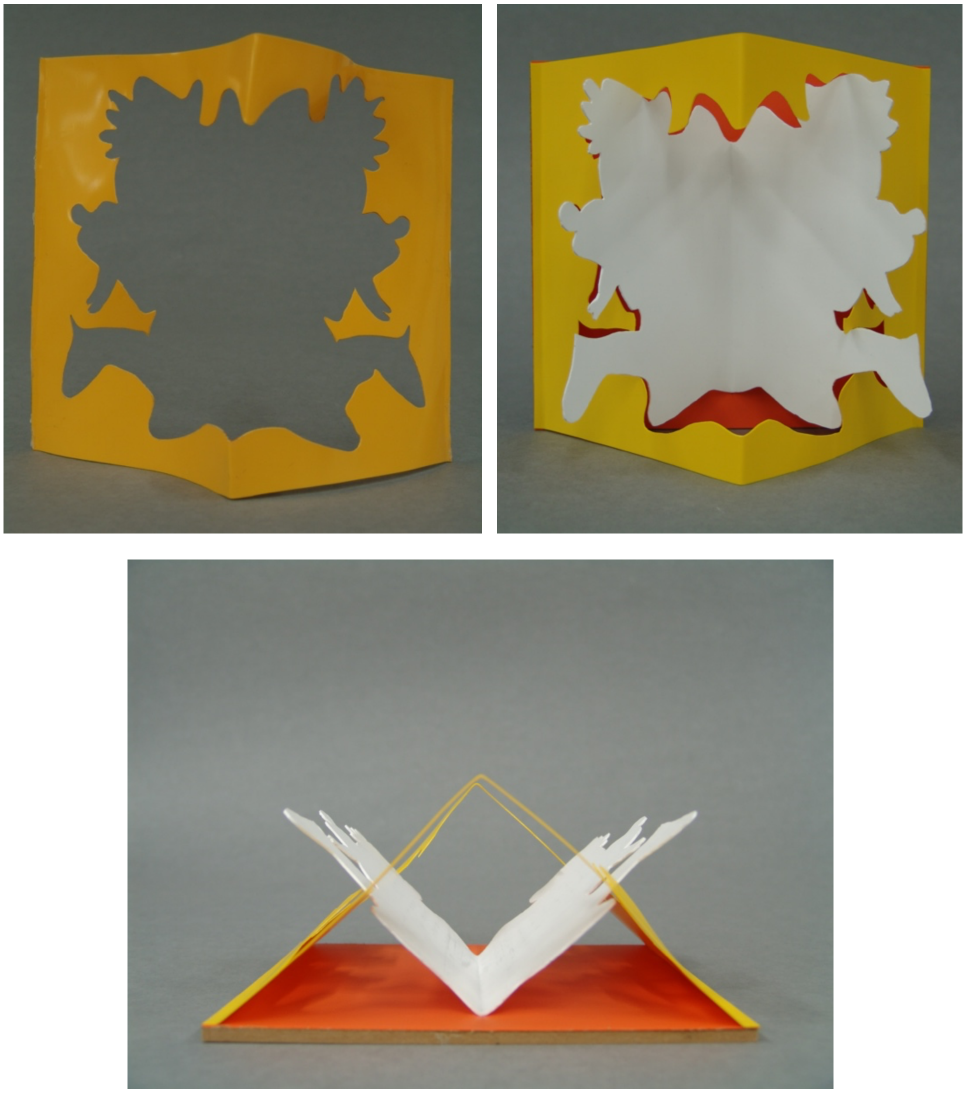

Scheme S1. A piece from the artwork *Dans la plage* that was used to prepare the replica with Mazzuchelli sheets. Photos by Sara Babo.

Following the procedure suggested in the article by Nunes et al. [4], calculating the degree of substitution (DS) of cellulose acetate requires measuring the absorbance at two different peaks in the infrared spectrum. One peak is the reference peak, which remains unchanged during degradation. The other is the probe band, which varies with degradation. In this case, the peaks are at 1030 cm^{-1}, associated with the vibration of the ether group of cellulose acetate (νCOC), and at 3330 cm^{-1}, related to the vibration of the hydroxyl group (νOH). The absorbance of these peaks is measured using the "peak height tool" in OMNIC. The ratio between these two values is then calculated. To connect this ratio to a specific DS, a calibration curve must be created based on cellulose acetate samples with known degrees of substitution, as shown in Table 1. The calibration curve plots the degree of

substitution against the ratio of absorbance at νOH to that at νCOC. The analyzed areas indicate the spectra are consistent, with the main point being the difference in intensity observed when comparing spectra on different faces of the same sample. The yellow samples show the least variation in intensity between the two faces. The calculated DS values seem to confirm the homogeneity suggested by the spectra. These samples were tested with several gels, which were then selected for use in the Escada pieces.

Table S1. Average and standard deviation of the degree of substitution for both faces of each sample analyzed from infrared spectra. For more details, see SM2.

Samples	DS average	DS standard deviation
orange 1	2.67	0.02
orange 1r	2.70	0.003
orange 2	2.69	0.02
orange 2r	2.72	0.04
orange 3	2.70	0.02
orange 3r	2.70	0.03
white 1	2.71	0.01
white 1r	2.72	0.01
white 2	2.73	0.01
white 2r	2.73	0.02
white 3	2.72	0.01
white 3r	2.71	0.02
yellow 1	2.71	0.02
yellow 1r	2.73	0.02
yellow 2	2.71	0.03
yellow 2r	2.73	0.02
yellow 3	2.69	0.01
yellow 3r	2.70	0.01

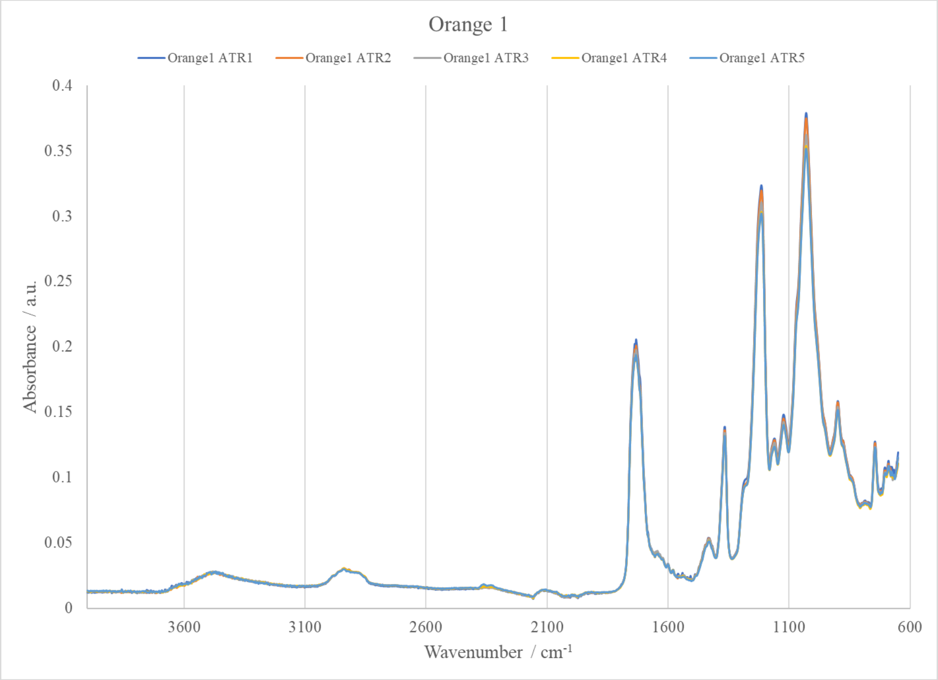

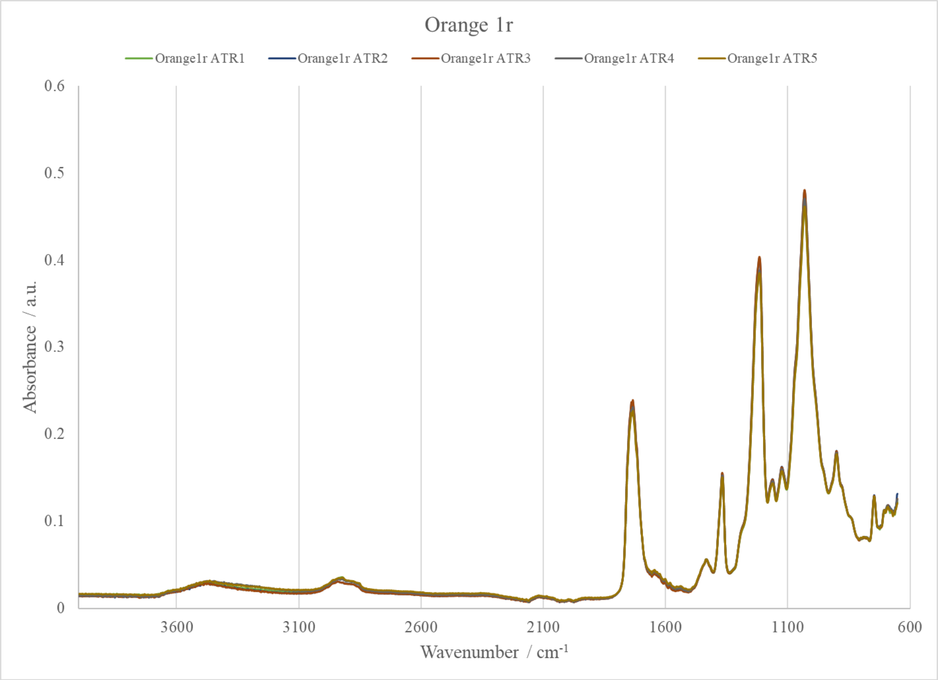

Figure S4. ATR infrared spectra of the ten spots analyzed on the orange 1 sample. The plot above shows the spectra of the five spots on one face, while the plot below displays the spectra of the five spots on the other face.

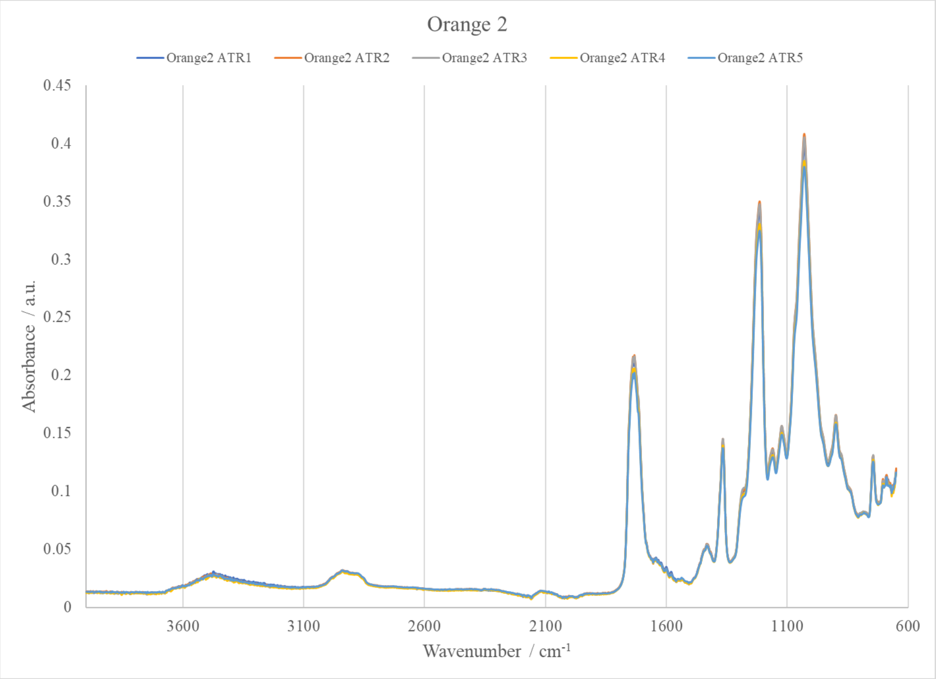

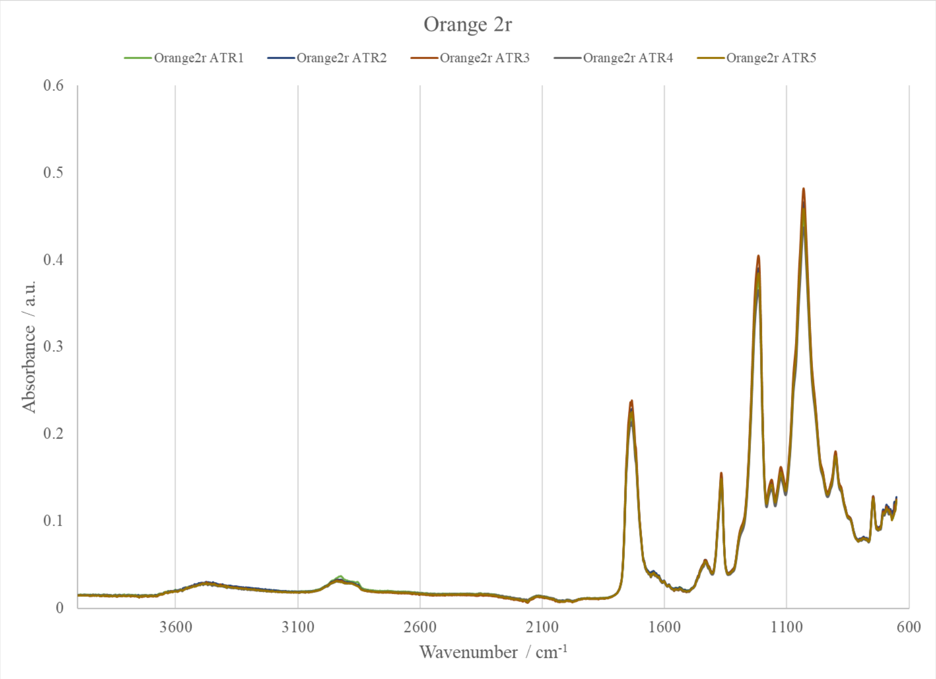

Figure S5. ATR infrared spectra of the ten spots analyzed on the orange 2 sample. The plot above shows the spectra of the five spots on one face, while the plot below displays the spectra of the five spots on the other face.

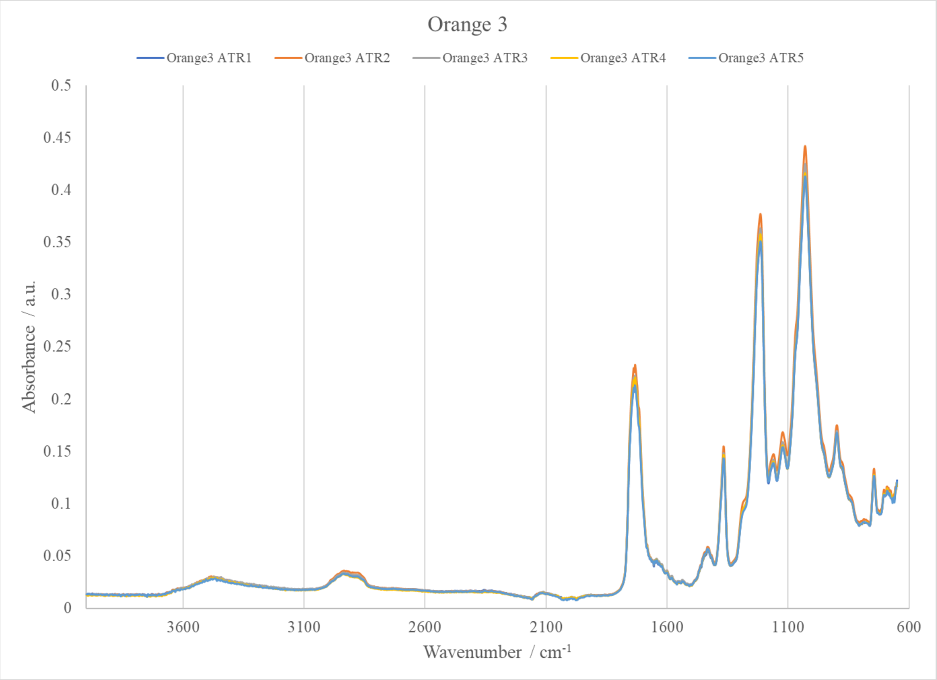

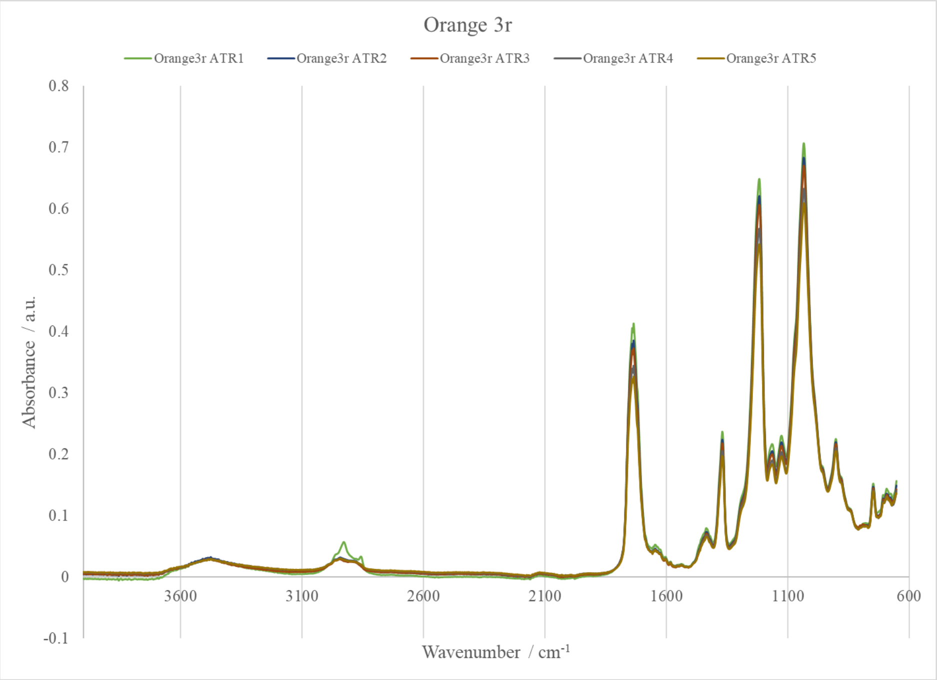

Figure S6. ATR infrared spectra of the ten spots analyzed on the orange 3 sample. The plot above shows the spectra of the five spots on one face, while the plot below displays the spectra of the five spots on the other face.

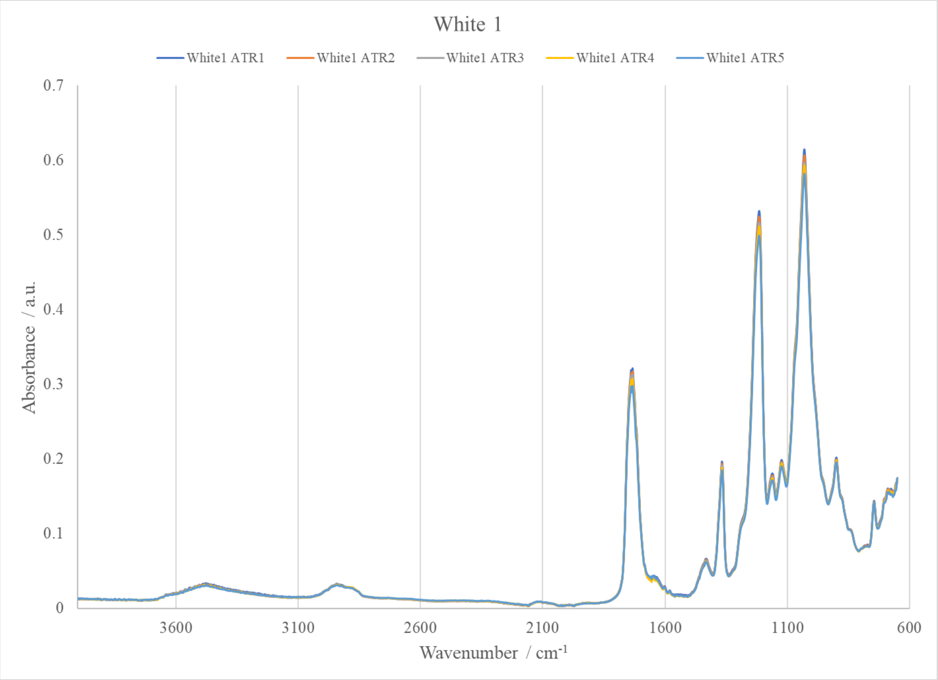

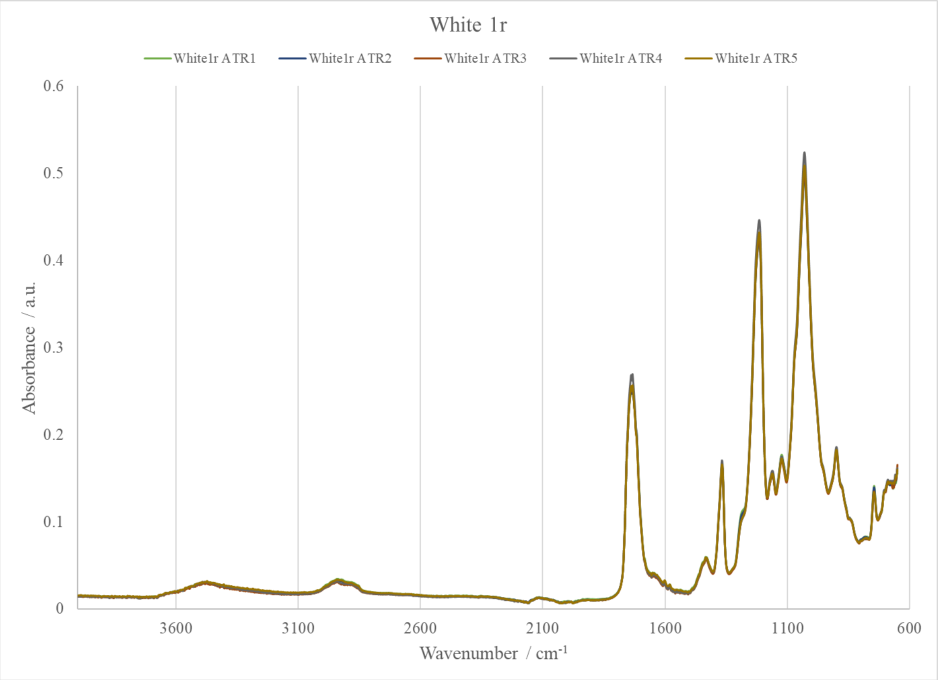

Figure S7. ATR infrared spectra of the ten spots analyzed on the white 1 sample. The plot above shows the spectra of the five spots on one face, while the plot below displays the spectra of the five spots on the other face.

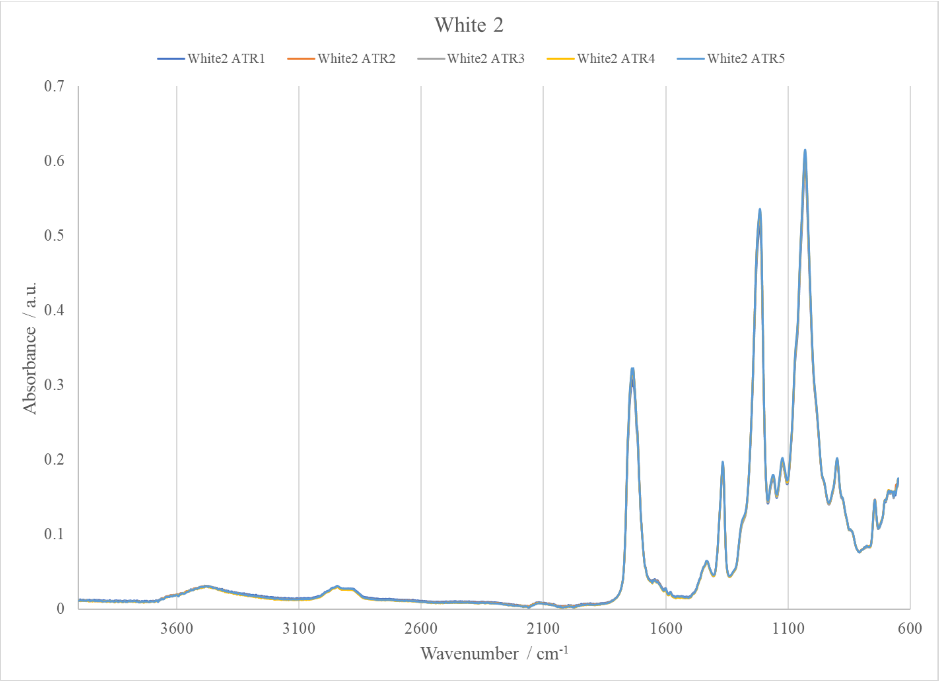

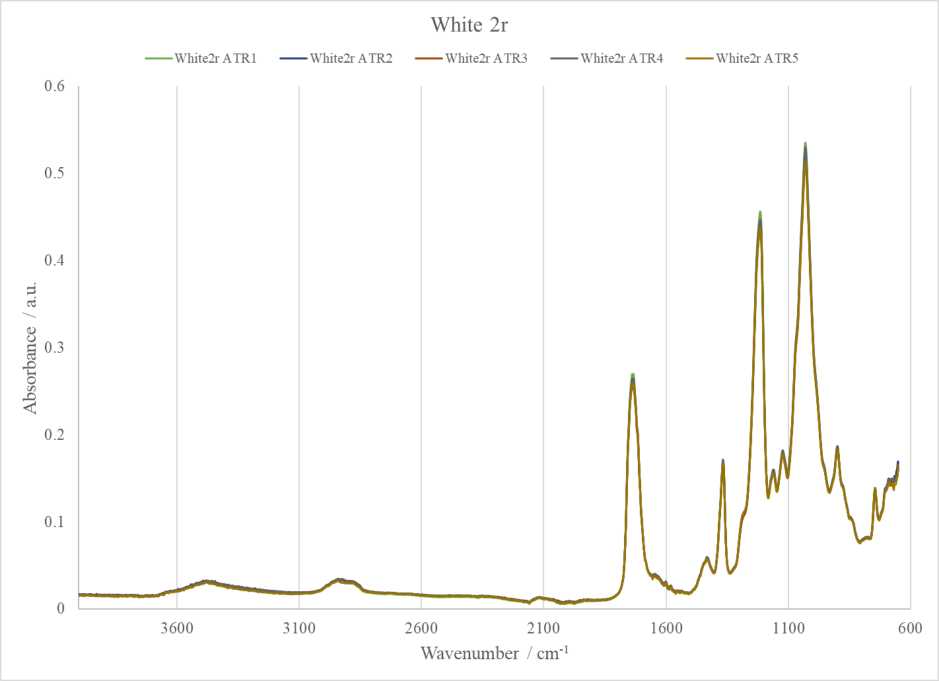

Figure S8. ATR infrared spectra of the ten spots analyzed on the white 2 sample. The plot above shows the spectra of the five spots on one face, while the plot below displays the spectra of the five spots on the other face.

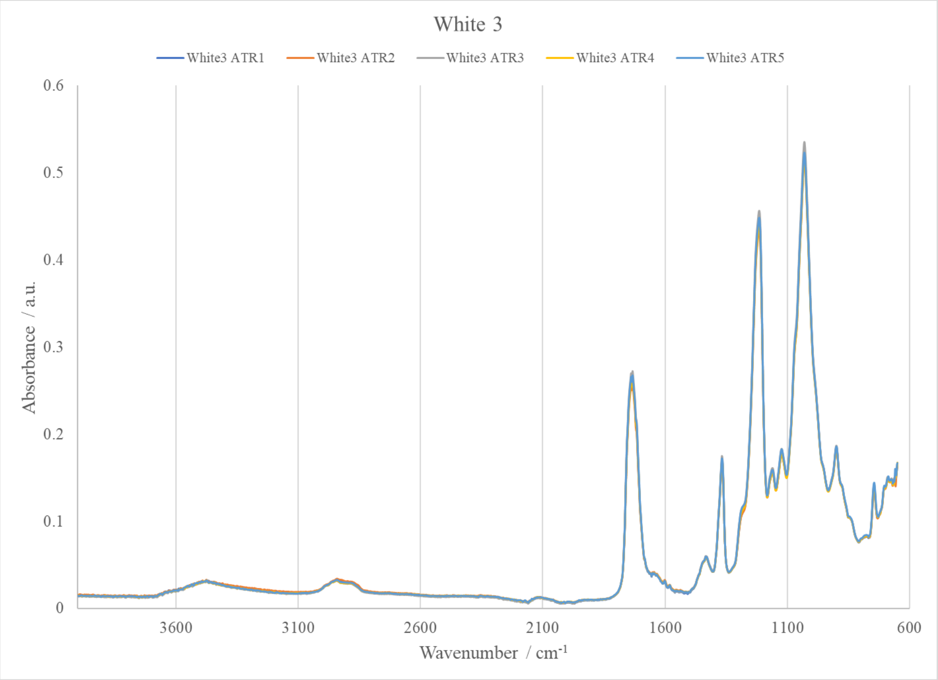

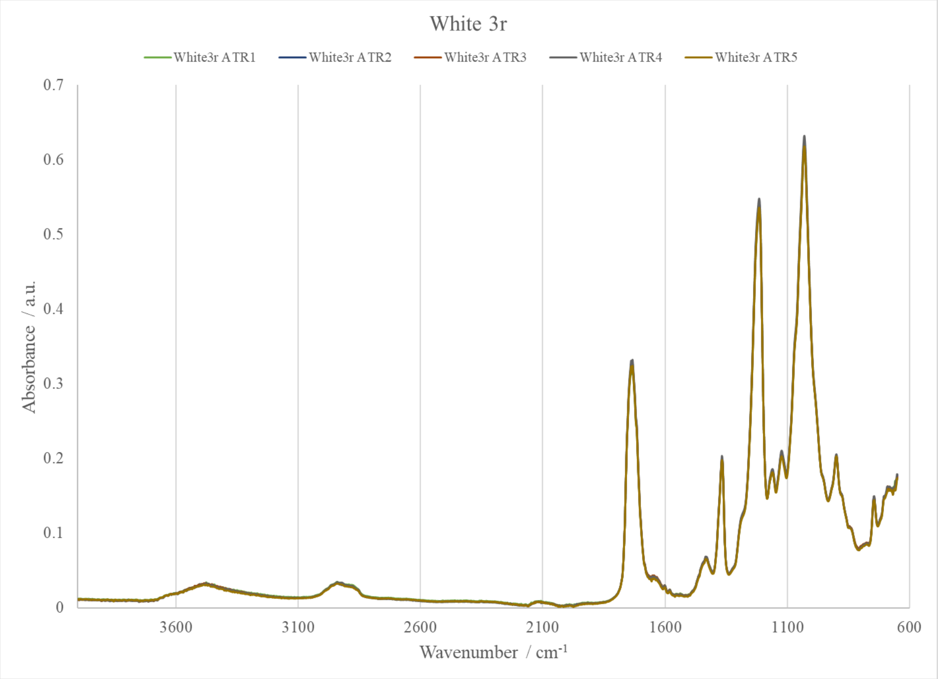

Figure S9. ATR infrared spectra of the ten spots analyzed on the white 3 sample. The plot above shows the spectra of the five spots on one face, while the plot below displays the spectra of the five spots on the other face.

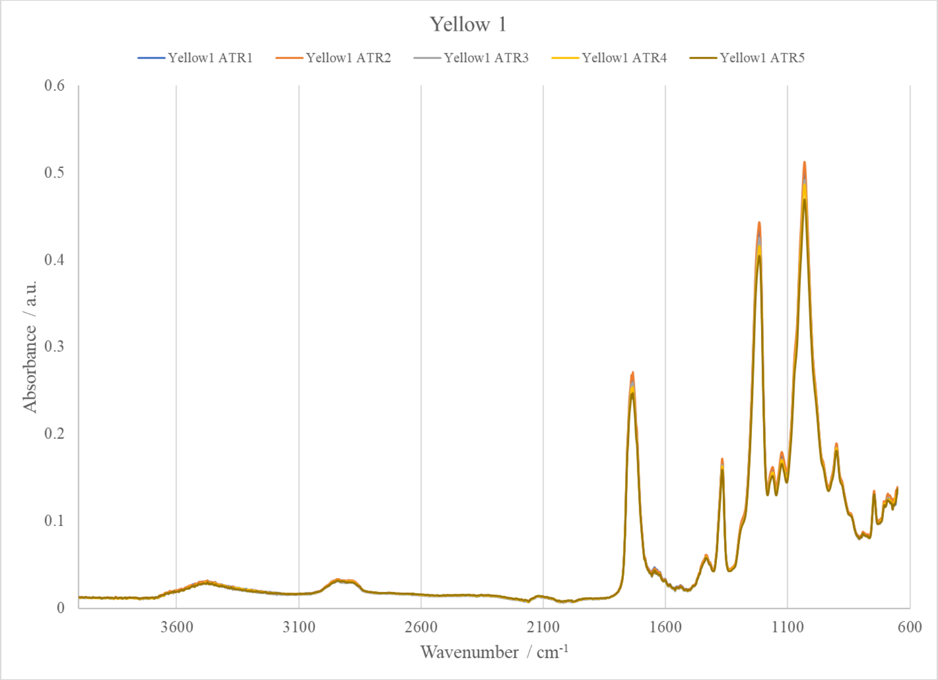

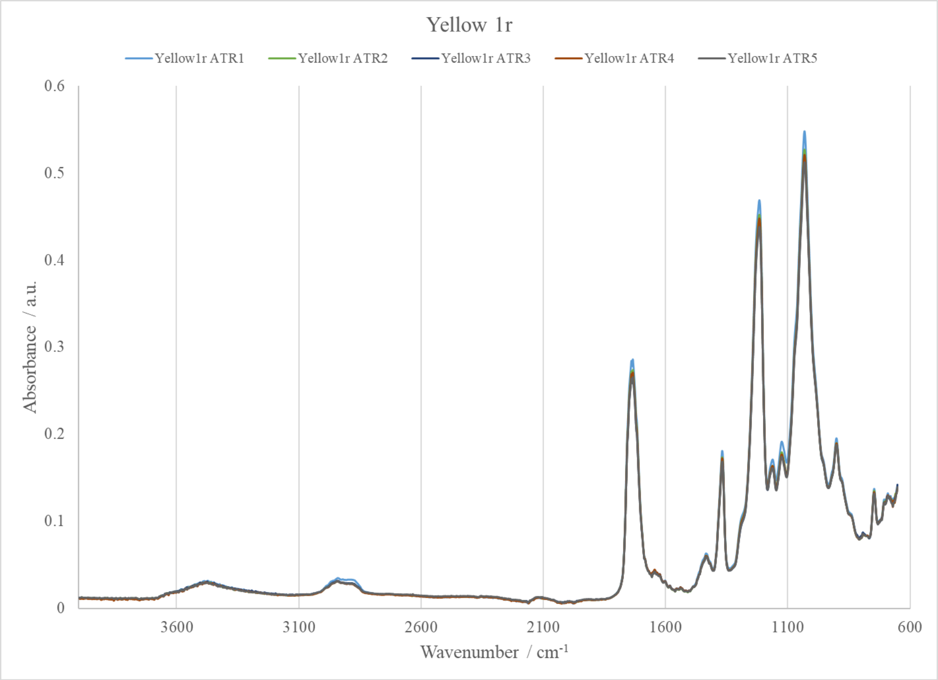

Figure S10. ATR infrared spectra of the ten spots analyzed on the yellow 1 sample. The plot above shows the spectra of the five spots on one face, while the plot below displays the spectra of the five spots on the other face.

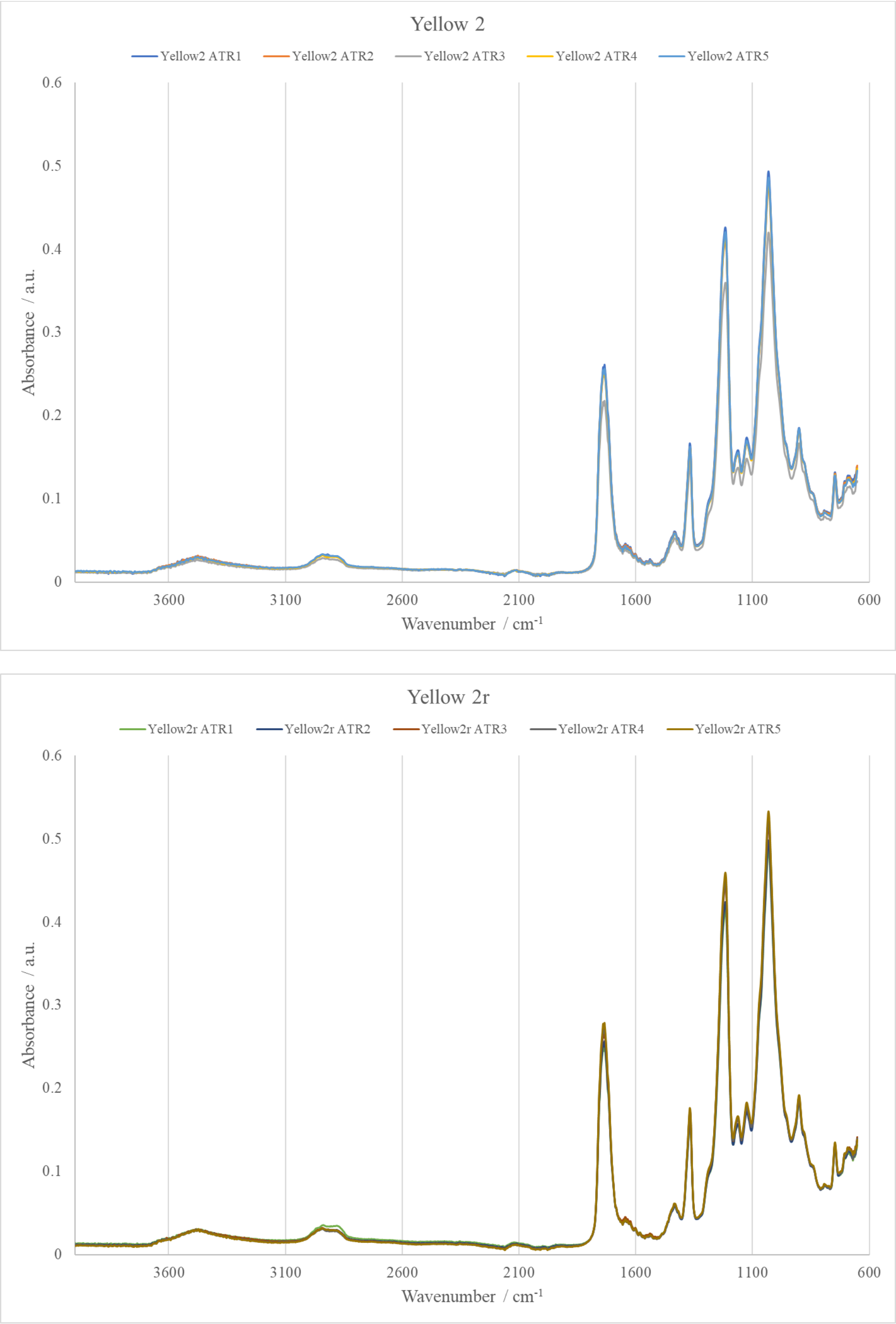

Figure S11. ATR infrared spectra of the ten spots analyzed on the yellow 2 sample. The plot above shows the spectra of the five spots on one face, while the plot below displays the spectra of the five spots on the other face.

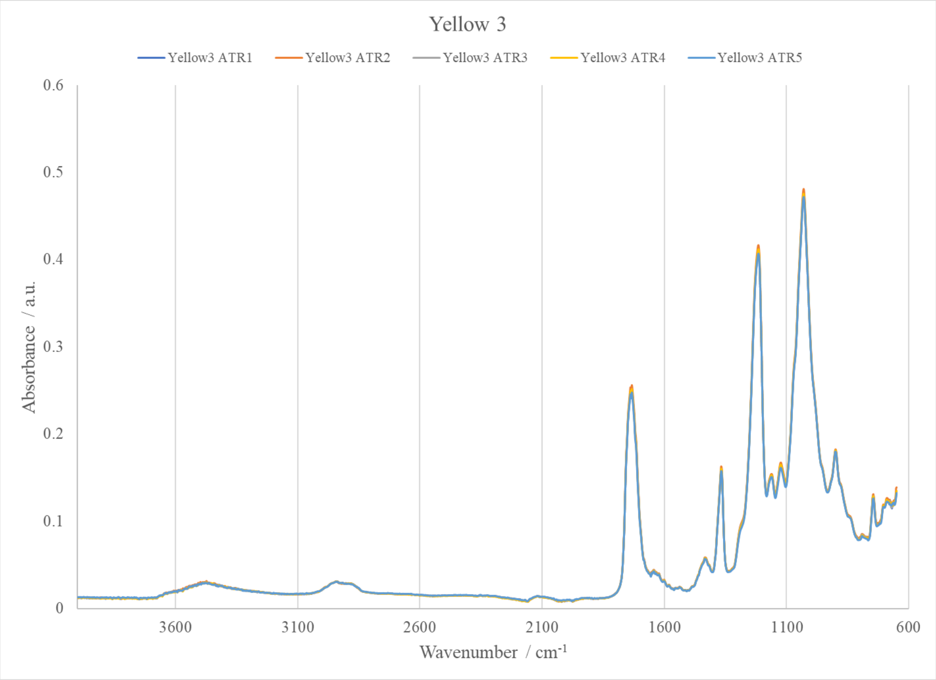

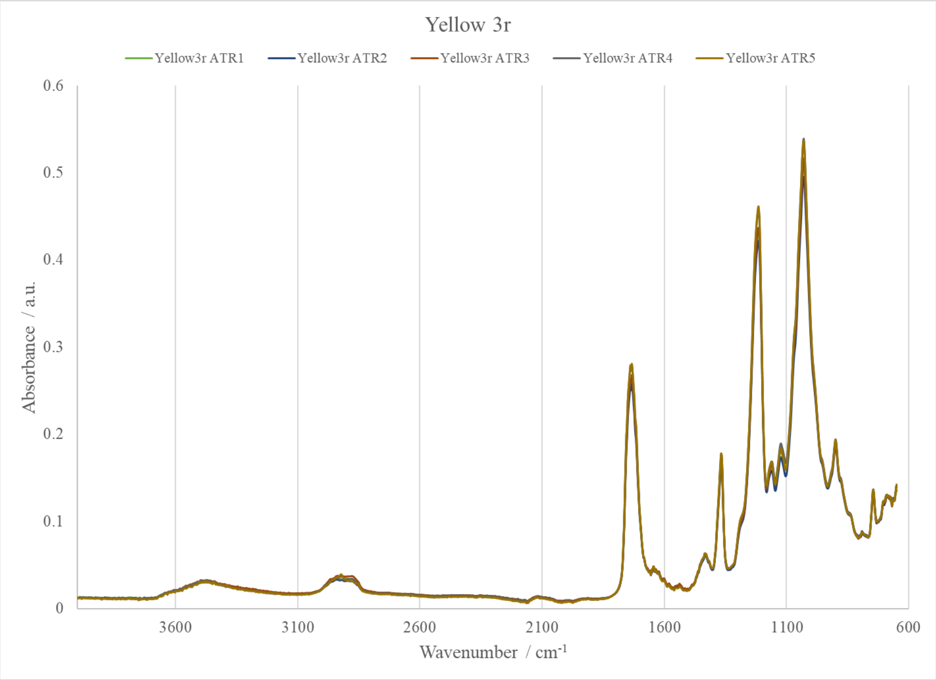

Figure S12. ATR infrared spectra of the ten spots analyzed on the yellow 3 sample. The plot above shows the spectra of the five spots on one face, while the plot below displays the spectra of the five spots on the other face.

Table S2. Degree of substitution for each point analyzed.

Spot	Degree of substitution
Orange1 ATR1	2.64
Orange1 ATR2	2.68
Orange1 ATR3	2.67
Orange1 ATR4	2.70
Orange1 ATR5	2.66
Orange1r ATR1	2.72
Orange1r ATR2	2.70
Orange1r ATR3	2.73
Orange1r ATR4	2.64
Orange1r ATR5	2.69
Orange2 ATR1	2.66
Orange2 ATR2	2.70
Orange2 ATR3	2.70
Orange2 ATR4	2.72
Orange2 ATR5	2.68
Orange2r ATR1	2.72
Orange2r ATR2	2.66
Orange2r ATR3	2.70
Orange2r ATR4	2.78
Orange2r ATR5	2.72
Orange3 ATR1	2.70
Orange3 ATR2	2.68
Orange3 ATR3	2.67
Orange3 ATR4	2.70
Orange3 ATR5	2.73
Orange3r ATR1	2.66
Orange3r ATR2	2.69
Orange3r ATR3	2.73
Orange3r ATR4	2.71

Orange3r ATR5	2.73
White1 ATR1	2.71
White1 ATR2	2.70
White1 ATR3	2.72
White1 ATR4	2.70
White1 ATR5	2.72
White1r ATR1	2.71
White1r ATR2	2.74
White1r ATR3	2.74
White1r ATR4	2.72
White1r ATR5	2.71
White2 ATR1	2.72
White2 ATR2	2.72
White2 ATR3	2.73
White2 ATR4	2.75
White2 ATR5	2.73
White2r ATR1	2.72
White2r ATR2	2.75
White2r ATR3	2.72
White2r ATR4	2.70
White2r ATR5	2.74
White3 ATR1	2.72
White3 ATR2	2.72
White3 ATR3	2.72
White3 ATR4	2.74
White3 ATR5	2.72
White3r ATR1	2.71
White3r ATR2	2.71
White3r ATR3	2.69
White3r ATR4	2.71
White3r ATR5	2.73

Yellow1 ATR1	2.69
Yellow1 ATR2	2.72
Yellow1 ATR3	2.73
Yellow1 ATR4	2.70
Yellow1 ATR5	2.73
Yellow1r ATR1	2.73
Yellow1r ATR2	2.75
Yellow1r ATR3	2.72
Yellow1r ATR4	2.69
Yellow1r ATR5	2.74
Yellow2 ATR1	2.68
Yellow2 ATR2	2.70
Yellow2 ATR3	2.74
Yellow2 ATR4	2.73
Yellow2 ATR5	2.68
Yellow2r ATR1	2.73
Yellow2r ATR2	2.74
Yellow2r ATR3	2.69
Yellow2r ATR4	2.75
Yellow2r ATR5	2.75
Yellow3 ATR1	2.70
Yellow3 ATR2	2.70
Yellow3 ATR3	2.67
Yellow3 ATR4	2.70
Yellow3 ATR5	2.70
Yellow3r ATR1	2.72
Yellow3r ATR2	2.68
Yellow3r ATR3	2.69
Yellow3r ATR4	2.70
Yellow3r ATR5	2.70

Supplementary Materials S3

S3.1 Raman spectra

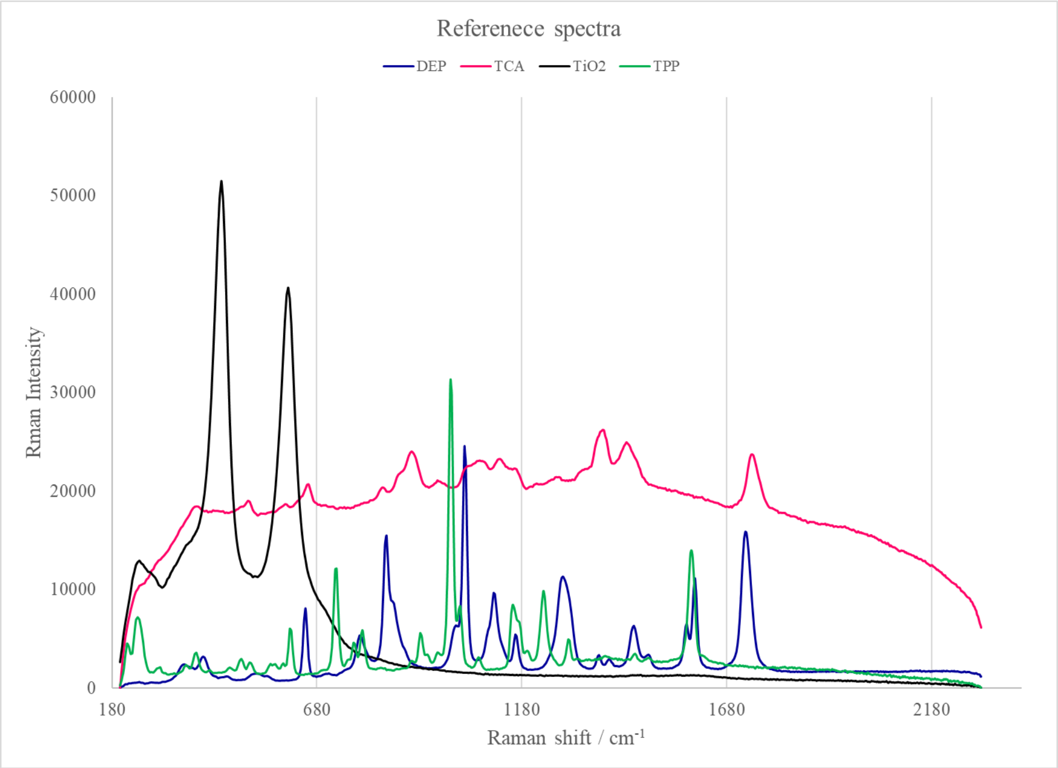

Figure S13. Raman spectra using Mira of the reference materials: cellulose triacetate (TCA), diethyl phthalate (DEP), triphenyl phosphate (TPP) and titanium dioxide (TiO_2).

Analyses were performed on a replica of José Escada's. Raman spectra. Three sheets of orange, yellow, and white cellulose acetate were examined using a portable Raman spectrometer, MIRA DS, and microRaman, described in section 3.2. By reviewing the literature and visually matching the spectra with reference spectra, it was possible to identify the peaks detected using NGSLab Spec. Peaks that were not immediately assigned with either technique were considered related to the colorants added to the cellulose acetate. The microRaman technique suggested the possible presence of a perylene compound used as the orange sheet's colorant, specifically a red one. The spectra obtained from microRaman are challenging to interpret due to high fluorescence. However, using the database integrated into the instrument's software for spectra acquisition and analysis, it was suggested there could be a match with PR179, a red colorant. As reported in the article by Vandenabeele et al. [36], to belong to this category, the colorant's spectrum should show peaks at 1500, 1400, 1175, and 1150 cm-1. It can be confirmed that this colorant is part of the perylene compound category, since, as noted in the article by Schulte et al. [37], these compounds feature peaks at 1300, 1380, and 1450 cm-1 related to stretching vibrations.

The pigment identified in the white cellulose acetate sheet—and possibly in the yellow sheet using MIRA DS analysis—is titanium dioxide in the rutile phase. The MIRA DS instrument also analyzed the cyanoacrylate-based glue on the surface of the orange cellulose acetate sheet. The data collected are described below. In Figure S13, the plot includes the Raman spectra of four reference materials: cellulose triacetate (TCA), diethyl phthalate (DEP), triphenyl phosphate (TPP), and

titanium dioxide in the rutile phase (TiO_2). DEP and TPP are common plasticizers used in cellulose acetate, while TiO_2 is a widely used white pigment.

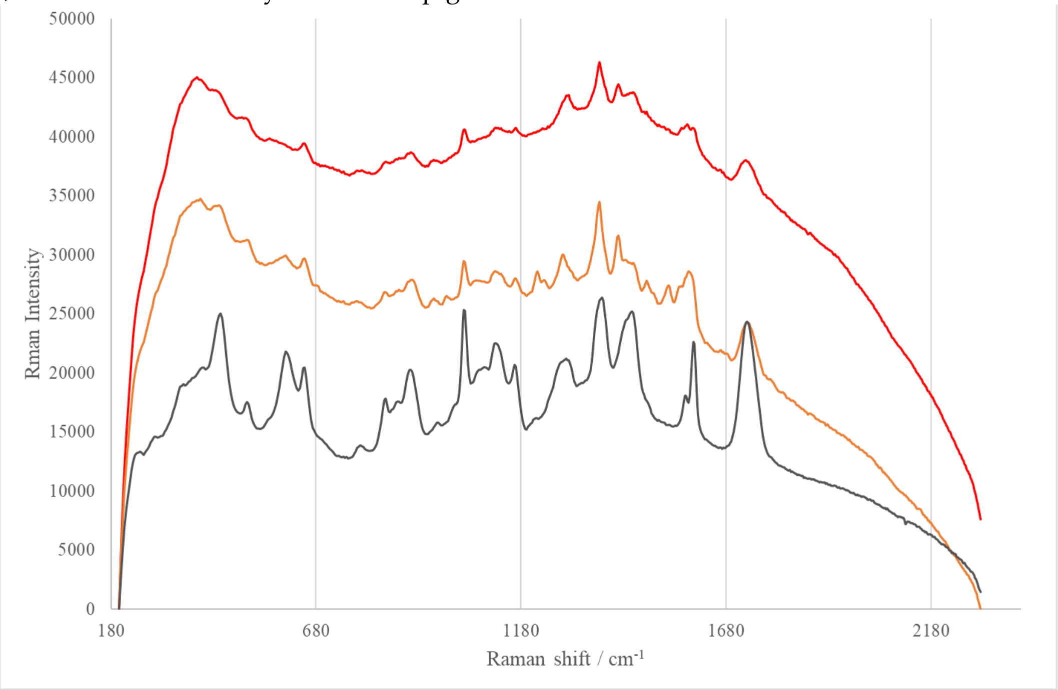

Figure S14. Raman spectra through MIRA related to the orange (red), yellow (orange), and white (black) cellulose acetate sheets. The acquisition times are 5s for the orange sheet, 8s for the yellow sheet, and 10s for the white sheet.

Figure S14 shows the plot with spectra, obtained using the MIRA DS instrument, related to the orange, yellow, and white cellulose acetate sheets.

In Table S1, a summary of the peaks detected using the program NGSLabSpec and their attributions can be found. The peaks identified from the analyses for the three materials, each represented by different colors, are related to various substances constituting the sheets. A technical paper from the Mazzucchelli factory [38], which supplied the materials, indicates that cellulose acetate has been combined with diethyl phthalate (DEP) and that no other plasticizers have been used. Based on this information, nothing further can be said about the colorants used, as the factory does not specify them. Another common plasticizer for cellulose acetate is triphenyl phosphate; however, since some of its major peaks—starting with the one at 1006 cm^{-1}—are absent in all three analyzed cellulose acetate sheets, its presence can be ruled out. According to the literature, peaks related to cellulose acetate that appear in all three materials include 652 cm^{-1}, which may correspond to the C-OH bond or the COO group in the acetyl group; between 910 and 914 cm^{-1}, associated with the C-H bond or the vibration of O=CO; between 967 and 977 cm^{-1}, linked to the C-O bond; 1117-1118 cm^{-1}, related to the vibration of the C-O-C glycosidic linkage; 1371-1377 cm^{-1}, corresponding to the symmetric vibration of the C-H bond and the bending of the methyl group; and 1728-1731 cm^{-1}, associated with the stretching of the carbonyl group (C=O). The last peak is observed in both cellulose acetate and the phthalate plasticizer. Regarding diethyl phthalate, its peaks are at 390-406 cm^{-1}, which can be attributed to the C-C bonds in the benzene ring; as well as at 652 cm-1, 780-788 cm-1, 849-850 cm-1, 1040 cm-1, 1117-1118 cm^{-1}, 1164-1167 cm^{-1}, 1283-1296 cm^{-1}, and 1450-1453 cm^{-1}, all associated

with the C-H bonds; and at 1580-1588 cm^{-1} and 1599-1600 cm^{-1}, characteristic of the benzene ring. The white pigment was identified as titanium dioxide in the rutile phase, with associated peaks at 251 cm-1, 286 cm^{-1}, 447 cm-1, and 606 cm^{-1}. Similar peaks were also observed at 443 cm^{-1} and 606 cm^{-1}. This suggests that titanium dioxide may be mixed with another colorant to produce the final color, though further analysis is needed to confirm this. The peaks, which, to this extent, have not found an attribution, could be considered as related to the colorants used.

Table S3. Orange, yellow, and white sheets with their attributions, compared to the reference spectra.

Cellulose acetate	Diethyl phosphate	Titanium dioxide	Orange sheet	Yellow sheet	White sheet	Attribution
		247			251	TiO_2
					286	TiO_2
	357				357	DEP
386	402		390	398	406	DEP [1], C-C benzene ring [2]
			427			colorant
		447		443	447	TiO_2
	460					
			499			cellulose acetate [1]
512				512	512	cellulose acetate
	534					
	559		567			DEP, colorant
603		610		606	606	cellulose acetate [1], COO [3], TiO_2
660	652		652	652	652	C-OH [4], COO [3], CO [5], benzene [2]
				679		colorant
	709					
	784		780	780	788	DEP
842	850		850	849	849	DEP [1], cellulose acetate
			882	882	879	cellulose acetate
911			911	914	910	C-H [4], ν_sO=CO [5]
974			967	967	977	C-O [4]
				998		colorant
	1019					
	1040		1040	1040	1040	DEP [1], C-H benzene ring, C-O-C [2]
1077				1071	1091	pyranose ring [4], νCOC [5]
1127	1111		1118	1117	1118	νC-O-C [4][5], C-H benzene ring [2]
1161	1164		1167	1167	1164	C-H benzene ring, ν(C-C-O) [2]
1216				1219	1216	νCO-C [5]
				1236		colorant
1264						
	1280		1296	1283	1290	νCO-C [5], C-H benzene ring [2]
1377	1368		1371	1371	1377	symmetric νC-H [4], δCH3 [5]
	1393					
			1417	1417		colorant
1435				1435		Asymmetric νC-H [4], δCH3 [5]

	1453	1453	1453	1450	C-H (DEP) [2]
	1489		1486		DEP
			1539		colorant
		1571			colorant
	1582	1585	1588	1580	DEP [1], benzene ring [2]
	1602	1599		1600	DEP [1], benzene ring [2]
			1667		colorant
1741	1725	1728	1731	1731	C=O stretching [1], [4], [5]

MicroRaman has been used to analyze the orange and white cellulose acetate sheets to determine the colorants. Due to the high fluorescence observed in the analysis of the yellow cellulose acetate sheets, this spectrum will not be shown. Figure S15 shows the area between 800 and 2000 cm-1 of the spectrum obtained from analyzing the orange cellulose acetate sheet, and Table S2 provides the attributions.

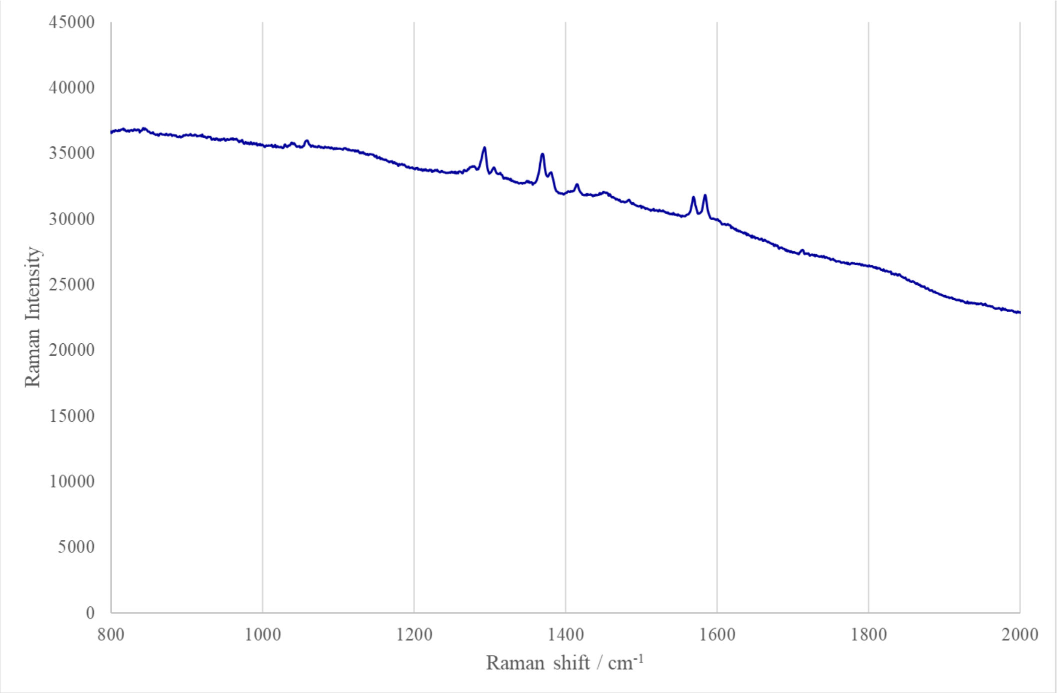

Figure S15. Raman spectra of an orange replica of *Dans la plage*, with the micro-Raman instrument. The acquisition time used was 15 seconds.

Table S4. Orange cellulose acetate sheet compared to the reference spectra of cellulose acetate and diethyl phthalate.

Cellulose acetate	Diethyl phthalate	Orange sheet	Attribution
	1040	1039	DEP [1], C-H benzene ring, C-O-C [2]
		1059	colorant
1077			
	1111		
1127			
1161	1164		
1216			
1264			
	1280	1293	νCO-C [5], C-H of the benzene ring [2]
1377	1368	1369	symmetric νC-H [4], δCH3 [5]
	1393	1381	DEP
		1415	colorant
1435			
	1453	1450	C-H (DEP) [2]
	1489	1483	DEP
		1569	colorant
	1582	1584	DEP [1], benzene ring [2]
	1602		
1741	1725	1712	C=O stretching [1], [4], [5]

S3.2. Raman description Handh

eld Raman spectroscopy

The Metrohm Instant Mira DS Raman spectrometer is equipped with a 785 nm diode laser with a maximum power of 100 mW, enabling data acquisition within a spectral range of 200-2000 cm^{-1}. This equipment provides a spectral resolution of 8-10 cm^{-1} and features a laser spot of 0.04 mm and a measuring spot diameter of 0.042-2.5 mm, depending on the working distance (distance from the probe to the sample surface), which can vary from 1 mm to 7.6 mm. For the present work, a working distance of 3 mm was preferably used, and the measuring spot diameter (measuring area) was approximately 1 mm. The detection technique used, known as Orbital Raster Scan (ORS), involves averaging the signal collected from relatively large sample areas (measuring spot) while maintaining the desired lateral resolution. All spectra were acquired with the maximum laser power and 10 scans, varying the integration time in the 200-30,000 ms range according to the target material and working distance. A minimum of three spectra were collected from the same sample to ensure data reproducibility.

Confocal micro-Raman spectroscopy

Confocal micro-Raman analysis was carried out using a Horiba Jobin-Yvon LabRAM 300 spectrometer, equipped with a diode laser providing excitation at 785 nm and a maximum laser power of 37 mW at the sample. The laser beam was focused through a 50× Olympus objective lens, resulting in a spot size of 4 μm. Laser power at the sample surface was kept between 9.5 and 0.37

mW. No evidence of paint degradation was observed during or after spectra acquisition. This system enables data acquisition in the 100-4000 cm^{-1} spectral range, with a 3 cm^{-1} spectral resolution. Spectra were acquired as a sum of 2-4 scans, with a 5-25s integration time. A minimum of three measurements were collected from the same sample to ensure data reproducibility, and a silicon reference was used for calibration.
